# Reactions, Reality, and Resilience in Adults with Crohn’s Disease: A Qualitative Study

**DOI:** 10.1093/crocol/otaf003

**Published:** 2025-01-16

**Authors:** Katherine Jones, Katherine Baker, Garry A Tew, Jenni Naisby

**Affiliations:** Warwick Clinical Trials Unit, Warwick Medical School, University of Warwick, Coventry CV4 7AL, UK; Department of Sport, Exercise and Rehabilitation, University of Northumbria at Newcastle, Newcastle Upon Tyne NE7 7YT, UK; Institute for Health and Care Improvement, York St John University, Lord Mayor’s Walk, York YO31 7EX, UK; Department of Sport, Exercise and Rehabilitation, University of Northumbria at Newcastle, Newcastle Upon Tyne NE7 7YT, UK

**Keywords:** Crohn’s disease, qualitative, health-related quality of life

## Abstract

**Background:**

Crohn’s disease (CD) is a lifelong condition that poses unique challenges. This study reports findings from a person’s perspective of living with CD to help enhance the understanding of an individual’s specific care and support needs.

**Methods:**

Semi-structured telephone interviews were conducted with a convenience sample of adults with Crohn’s disease recruited from Newcastle Hospitals NHS Foundation Trust. Data were analyzed after data collection using thematic analysis.

**Results:**

Forty-one (68% female) participants aged 49.1 ± 12 years with a disease duration between 1 and 55 years were interviewed. Three overarching themes emerged, along with 12 subthemes: (1) reactions to presenting symptoms, emotions, and challenges at diagnosis; (2) reality of living with the condition, seeking information, decision making, psychological challenges, experiencing symptoms/complications during remission and the impact on social life, education, employment, and relationships; (3) Resilience involving emotional adaptations, strategies on self-management, social comparisons as a means of coping and barriers to resilience.

**Conclusions:**

The results highlight the complex health journey and challenges faced by people living with Crohn’s disease and provide health care professionals with a greater insight into the psychological challenges and emotional complexities of the condition to facilitate a more holistic approach to planning care.

## Graphical Abstract

**Graphical Abstract:**
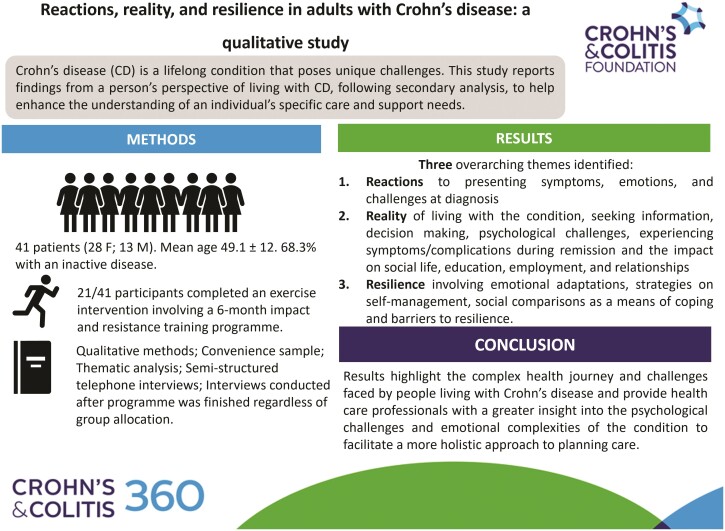

## Introduction

There is a growing body of qualitative research emerging that explores a person’s perspectives and challenges of living and managing inflammatory bowel disease (IBD), the collective term for CD and ulcerative colitis (UC).^1–5^ The main themes emerging are the unpredictability of the disease, the emotional turmoil associated with symptoms, the strive to maintain a normal life, expecting more information about their disease process, shared decision making, and symptom management strategies. However, many of these studies did not distinguish findings between CD and UC, despite having clinical differences and features; disease behavior (eg, structuring, penetrating, perianal), complications (eg, fistulas, abscesses, and strictures), disease treatment and response, and higher prevalence of extraintestinal manifestations.^6–9^

We recently reported the results of (1) a randomized controlled trial (RCT) PROTECT (PROgressive resistance Training Exercise and Crohn’s disease Trial), which investigated the effect of a 6-month combined impact and resistance training program on bone density and muscular function in adults with inactive to mildly active CD.^10^ (2) Following completion, a qualitative exploration of exercise experiences and perceptions in adults with CD was undertaken to investigate views and values and improve understanding to support the development of self-management options.^11^ Closer analysis revealed greater insights that warranted further exploration.^12^ Therefore, this study reports the findings from a person’s perspective of living with CD to help enhance the understanding of an individual’s specific care and support needs.

## Materials and Methods

### Study Design and Sampling

PROTECT (PROgressive resistance Training Exercise and Crohn’s disease Trial) was a 6-month RCT designed to investigate the effect of a 6-month combined impact and resistance training program on bone density and muscular function in adults with inactive to mildly active CD.^10^ A convenience sample of 43 participants who had taken part in PROTECT, regardless of group allocation were invited to take part in a semi-structured interview. Of these, 2 participants declined to participate in the interview. A qualitative research method was employed with a primary aim to explore the exercise perceptions and experiences of PROTECT, published elsewhere.^11^ Data analyzed after data collection aimed to explore disease history, including diagnosis, symptoms, and disease management of participants. Interviews were undertaken by a member of the trial team (KJ) who had delivered PROTECT, a 6-month intervention, thus a rapport had been established prior to conducting the interview. Having a good rapport with participants allowed for the generation of rich data, better information, and data access due to the trust and understanding built.^13,14^

### Data Collection

A total of 41 participants (*n* = 21 exercise; *n* = 20 control) took part in the individual semi-structured telephone interviews (25–45 min duration) following the completion of the 6-month program. The interview guide was based on previous qualitative research.^15^ All interviews consisted of open-ended questions (Supplementary Material 1). All telephone interviews were audio recorded and later transcribed verbatim. Participant identification numbers were used throughout to maintain anonymity.

### Ethics

Ethical approval was granted by NHS Newcastle and North Tyneside Research Ethics Committee (Ref: 17/NE/0308) and Northumbria University Ethics Committee (Ref: 656). Written informed consent was obtained prior to each interview.

### Data Analysis

Data were transcribed verbatim and analyzed using Framework analysis,^12,16^ a method used to interrelate steps to assist the management of qualitative data and analysis.^12^ This framework method was flexible, and not aligned with a particular theoretical approach so as to be adaptable for the approach to generate themes.^12^

Two researchers (K.J. and J.N.) familiarized themselves with the data by repeatedly reading each transcript and coded the first 6 transcripts independently and a thematic framework was developed. The 2 researchers then met to compare notes, discuss the codes, and refine the thematic framework. This process ensured that participants’ perspectives were captured by 2 researchers, both of whom had different backgrounds: exercise physiology (K.J.) and physiotherapy (J.N.). The remaining transcripts were shared, and the thematic framework was applied. The framework was flexible to allow new codes to emerge. The 2 researchers met frequently to discuss any new codes, which were applied to the framework and earlier transcripts. Disagreements were resolved through consensus or discussion with a third author (G.T. or K.B.).

To explore the full dataset and facilitate data interpretation, data charts were developed into a Framework Matrix. Data mapping of similarities, differences, and connections was conducted independently by 2 researchers. The 2 researchers met frequently to develop and refine themes, with both perspectives considered and captured in the analysis. The final themes were discussed with the coauthors (G.T. and K.B.) and then further refined.

## Results

Forty-one participants took part in this qualitative study. Participant and individual characteristics are presented in Table 1 and Supplementary Material 2, respectively. Of these participants, 68.3% were female (*n* = 28) with a mean age of 49 ± 12 years; 43.9% were employed full time and all were of white ethnicity. The median duration since diagnosis was 204 months (IQR 60-383) and most had an inactive disease (68.3%; Crohn’s Disease Activity Index < 150).

**Table 1.** Participant characteristics (*n* = 41).

Age, mean (SD), years	49.1 ± 12
Sex, *n* (%)	
Female	28 (68.3)
Male	13 (31.7)
White ethnicity, *n* (%)	41 (100)
Employment status, *n* (%)	
Employed full-time	18 (43.9)
Self-employed	5 (12.2)
Unemployed	6 (14.6)
Retired	8 (19.5)
Age at diagnosis, median (IQR), years	31 (23-37)
Duration of diagnosis, median (IQR), months	204 (60-383)
Disease activity, mean (SD)	
Fecal calprotectin, μg/g	52 (41-98)
CDAI	101 (65-158)
CDAI activity status, *n* (%)	
Inactive (<150)	28 (68.3)
Mildly active (=150-219)	13 (31.7)
Surgical history^a^	
Stoma	6
Resection	16

Abbreviations: CDAI, Crohn’s Disease Activity Index; IQR, interquartile range.

^a^Multiple answers possible.

Three overarching themes were identified through thematic data analysis: (1) reactions, (2) reality, (3) resilience. Direct quotes with reference to the participants’ identification number are presented to illustrate key and sub themes, further quotes are provided in Supplementary Material 3.

### Reactions

#### Early symptoms

Fatigue, abdominal discomfort, joint pain, diarrhea, and weight loss were commonly reported as early symptoms; however, many participants only sought health care advice when bloody diarrhea, increased toilet frequency, or urgency accompanied this. Some only sought healthcare advice after being encouraged or “forced” by a family member or friend. Participants also recalled delaying seeking healthcare advice, with some experiencing early signs of Crohn’s disease but often expected their symptoms to improve, did not see them as severe enough, or believed these to be as a result of: “growing pains,” “sitting/lying funny,” “stress,” “old age,” “something I’d eaten,” or “period cramps.” This led to individuals self-diagnosing and adjusting to a new “norm” that included experiencing abdominal discomfort, joint pain, and tiredness.

I just thought it [joint pain] was normal because I was quite young and you get told you have growing pains, but then the blood came, and my parents dragged me to doctors (R031)

I would never go to the doctor and be like oh I’m tired I just feel like you would be laughed out the door…maybe because it’s not like life threatening and I just feel like maybe I was wasting their time, plus I just had a child so just assumed it was that and stress (R045)

… I was quite surprised about being diagnosed because I just felt mainly fatigued and a bit sick at times but I thought I had a slight tummy bug … now I think about it I did have symptoms for a long time but guess it was only when I felt they were serious did I speak to my GP (R033)

#### Challenges at diagnosis

When healthcare advice was sought this was through a general practitioner (GP) appointment or directly through admission to accident and emergency (A&E). However, the answers given were not always specific or helpful. Some participants recalled difficulties in communicating presenting symptoms to GP’s and not being listened to in A&E. Participants described accusations of attention-seeking behavior, severity of symptoms not being acknowledged, or been given tablets as a way to “get rid” of them. The recurrence or worsening of symptoms caused participants to revisit their GP to seek answers, which, in some cases, led to receiving a misdiagnosis of irritable bowel syndrome and severe constipation. In contrast, for others, this led to a gastroenterology referral and information support from their GP. Many participants had sympathy for GP’s, acknowledging the time pressures they are under and the complexities of the condition, for example, fluctuating symptoms, and presenting differently between individuals. However, they noted that they would not have recognized this at the time of diagnosis and can only now see the complexities the disease presents.

… I just remember having really bad abdominal pain like nothing that I have ever experienced before so my wife took me along to A&E and they, they, they just fobbed me off (R036)

I went back to the doctors who basically said what do you want me to do about it, and I just said I wanted to know what was wrong with me (R002)

I wouldn’t necessarily say angry because they [GP’s] do so much, but I could have had years on better medication and been able to do more instead of feeling the way, I just knew something wasn’t fully right (R038)

#### Emotions around diagnosis

Prior to receiving a diagnosis many expressed feeling anxious, worried, helpless, frustrated, and overwhelmed because of the uncertainties, undergoing multiple medical tests, and fearing that they may have a life-threatening condition. Many participants also recalled feeling distressed following the use of diagnostic tools such as endoscopies, particularly. This, in turn, led to a few participants becoming scared to return to a hospital, or even refusing to have these procedures again.

Never heard of it but I mean I was more concerned as to what like what it was and whether I was gonna die from it to be fair. I’d just had me son and didn’t need this (R045)

…once I was released from hospital after being diagnosed I kind of, I kind of didn’t go back to Dr’s for about two years because I was a bit frightened about all the tests and things (F018)

Some of these emotions continued immediately after receiving a diagnosis, with some participants acknowledging feeling in shock and denial. These emotions were particularly apparent in those who required immediate surgery, hospital admission, or commencement of intravenous biologics. For some, because the condition was not something they had heard of before they recalled feeling vulnerable as they were unsure of the possible impact the disease would have and if it was treatable. For those who had heard of the condition, emotions varied, some felt reassured knowing the experience of a close family or friend “doesn’t take any medication and are fine,” while others felt frightful knowing and seeing what their close friend or family had been through.

Going from not having anything to being diagnosed and then needing a stoma just was a lot to take in (R046)

I’d heard about it with my line of work but me myself would never have thought that, because people with Crohn’s are poorly, quite poorly and I didn’t feel like that (R033)

### Reality

#### Information seeking

Shortly after receiving a diagnosis many participants described struggling with the condition. Some described feeling in “limbo” around getting answers, relating to causes, treatment, and management. Knowledge was sought through the internet, charity organization forums, and social media to educate themselves about the condition. For some, this evoked sadness and stress whereas for others they saw it as a positive way to learn about their condition. Some participants mentioned the use of local support groups as a means of having “proper information” from the right sources such as healthcare professionals. Information seeking continued for a lot of participants who explored alternative therapies such as oak bark, homeopathy, reflexology, and massages. Some participants also sought additional advice from healthcare professionals and online resources when it came to having a family, with concerns around the hereditary nature of the disease.

I initially had a look myself and then was told it was not hereditary because I thought I wouldn’t have children if it was hereditary. They said it wasn’t (F009)

I’ve tried various alternative therapies, I would try anything to see if it would work, I even tried a herbalist and oak bark, but I think my intestines were so badly damaged at this point that I don’t think there was anything that was actually going to help (R005)

#### Decision making

For the few participants who had a stoma, the majority felt it was their last treatment option as “a battle that I was losing” and others “didn’t have that option to try things” due to requiring emergency surgery. Despite this, all participants agreed that it was the right option and gave them “freedom” and a “better existence.” Interestingly, some participants were very against having surgery, particularly a stoma, after “hearing stories” and reading online, and saw experiencing medication side effects or disease symptoms as a better option. A few participants discussed the potential of having surgery or a stoma as a future option that they may require, using online resources and discussions with IBD nurses to develop their knowledge and understanding to help them feel prepared. A lot of participants discussed the side effects they experienced from medication as being manageable but some which were “worse than the disease.” However, no one mentioned the use of online resources to help them make their decision around taking medication or commencing IV drugs. Some acknowledged they would use the internet to look at side effects but were happy their consultant provided multiple options.

I knew I was at that point where I probably needed it, was it something I wanted, no, but was it something that was going to give is [me] any sort of life, yes (R030)

I do expect to probably have surgery in the future, I’ve done my reading and the surgery is my last option, but we’ll be prepared for it (R032)

If my medications may need to change, she will give me a few options and ask me what I would rather do and you know I really like and I really appreciate her letting me make decisions (R015)

#### Psychological challenges

All participants discussed their individual struggles of living with an unpredictable disease. However, the impact the disease played on mental well-being was extremely apparent, with participants describing it as a “psychological battle.” The impact on confidence and increased levels of anxiety were often discussed, primarily due to fear of accidents, taking public transport, or going to unknown places. Medication side effects also played a part in impacting confidence and increasing anxiety, with many recalling gaining a substantial amount of weight, with some describing this as a reason they did not want to leave the house. Some also thought the medication they had been given had caused other health problems. Self-identity was also mentioned by a few participants who described losing their way and felt the disease “stripped” them of who they were.

…it just takes a grip and doesn’t give you a break it can very easily er drag you down, that’s what I’ve struggled with most with my condition (R036)

I had to reduce them [steroids] down until I stopped taking them, made is [me] gain loads of weight, helped my bowels but not what you want before your mate gets married (F019)

It’s the worst thing that I’ve ever had in me life it changed who I was you know, I’m a confident person and was a confident person before all that happened and it completely stripped me of who I was (R015)

#### Ongoing symptoms and complications

Experiencing symptoms or disease-specific complications during periods of remission was discussed by many participants. Although most discussed fatigue and tiredness during active disease periods, a lot of participants continued to feel like a “walking zombie” with “constant brain fog” even during periods of remission. Participants described this as extremely debilitating, felt misunderstood, and not taken seriously. Some discussed the need to nap, rest, or pace themselves, however, most found nothing helped. Joint pain was another symptom discussed; however, some participants were unsure if this was down to “age” or “menopause” and not the disease, whereas others knew it was an indication their condition was flaring up. Many participants discussed using handwarmers, avoiding repetitive activities and rest as a way of relief. Some recognized that symptoms of fatigue or joint pain were not often discussed during hospital appointments.

I’m just constantly in a fog, honestly sometimes I would prefer to have the pain or the diarrhoea because you know that goes away (R038)

…there’s no over the counter medication for tiredness or fatigue it’s just constant and I haven’t found anything that particularly helps yet and I’ve had the disease over 30 years (R028)

I get joint pain…I know it’s related to the condition but I’ve never been treated specifically for it, I guess thinking about it now it’s not something that you get asked about (R048)

Like fatigue, the presence of abdominal pain was largely discussed when the disease was active and interestingly before any surgical intervention. Although some participants experiences abdominal pain during periods of remission, the cause was often as a result of stress or following a meal. The majority of those who had surgery described not experiencing any abdominal pain at all, and if they did these were “niggles.” When abdominal pain was present many participants discussed using hot water bottles, having a hot bath, waiting for it to pass, drinking fluids only or sleeping as a way of managing the pain. Pain relief was also used; however, a few participants mentioned they avoided using some medication due to it making them drowsy or adversely impacting their day-to-day activities.

…generally I just deal with it [abdominal pain] and I’m sort of in that mindset it’s part of the disease and hopefully that it will go soon (F022)

Before I had the surgery it was kind of constant…never had a break with it…I don’t get abdominal pain at all now, I’ve rarely had that since the surgery (R030)

Being a writer and writing books is really hard to do when you’re in pain or when your drowsy from the medication so I try not to overdo it (F002)

Some participants also discussed the “vicious cycle” with the disease-specific complications, experiencing fistulas, abscesses, and fissures and others recalled it was “just one thing after another” with the development of extraintestinal manifestations such as arthritis, ankylosing spondylitis, psoriasis, and osteoporosis. Few participants knew these were associated with the condition; however, for others, it came as a surprise.

You have residual problems like joint aching, rubbish skin, dry eyes and I don’t think it’s until somebody tells you it’s part of the condition that you actually know (R009)

…people say oh you’ve got Crohn’s but then its abscesses, its fistulas, its fissures, all of which I’ve had. Then there’s arthritis problems, it’s just one thing after another and you have to find out for yourself (R024)

You get use to having the disease like you adapt but there’s always something that comes out that surprises me … I have COPD and arthritis how can they be linked to the bowel you know (R005)

#### Employment, Education, Social life, and Relationships

Subsequent strains on marriage, social life, education, and jobs were also highlighted by participants as being impacted because of the condition. A few participants put the condition down to causing their marriage to breakdown because of lack of understanding or not being empathetic. Participants described the social implications of living with the disease, often missing out on social events and activities primarily because of others not understanding the condition, not wanting to discuss their symptoms or fear of embarrassment.

I think it personally was the reason my first marriage didn’t work, it just completely broke down with me always being ill and him not understanding when I said I was unwell (R015)

There’s been times where I’ve missed events or parties and stuff because of it or even because I’d be scared that I got ill when I was out the house (R045)

A few participants discussed their difficulties with employers particularly when experiencing a flare-up; some made the decision to become self-employed to control when and where they could work, some chose early retirement and one participant described having to change careers due to the development of a secondary complication (rheumatoid arthritis) that meant they could no longer do their job. Participants also recall their concerns surrounding job security and money worries should they require sick leave. Support from employers was discussed by a few participants including the provision of a keyboard roller ball for joint pain, flexible hours for fatigue/hospital appointments, and the ability to work from home if required. Conversely, some participants concealed their condition from work colleagues, friends, and some family members due to feeling scared or embarrassed.

I told my boss when I need to be off for appointments, but I haven’t told anyone I have Crohn’s, not even my friends, no one apart from my husband and my two sons know. It’s just embarrassing… I just don’t want people to know that, I wouldn’t have told my boys if I wasn’t unwell (R016)

I actually went self-employed when I started falling asleep at my desk at work which you can imagine wasn’t the best impression I could give (R032)

I mean I stopped working a few years back because I felt as though it sort of won and having the ups and downs with it and the stress from work not understanding (R038)

For those participants diagnosed during childhood or teens, mental struggles watching others achieve their goals and lack of support/empathy received from lecturing staff or schools were recalled. Some participants described their school being accommodating and supportive.

It was very mentally exhausting seeing people your age go off and celebrate finishing uni and getting jobs and you’re stuck in the hospital or at home because of this…. you lose friends, you stop getting invited to places or events because you aren’t well and that takes it toll (R018)

I missed a lot of my school years, the teachers at my first school weren’t really understanding but at my second school they were a lot better but think at that point I was in hospital a lot so they couldn’t really say anything (R013)

### Resilience

#### Emotional adaptations

Resilience-related coping was widely discussed by participants, as a means of obtaining knowledge from living with the condition to improve disease control and management. Participants described how their emotions towards their initial reaction to the disease and living with the disease had changed because of acceptance. For example, for some this meant appreciating what they have, adapting to lifestyle changes, learning about the condition, being aware of potential challenges or limitations and for others this was recognizing it was important to have a “positive outlook.” Disease acceptance was evident in those with a longer disease duration or who had undergone a surgical procedure. These participants reflected on trying to find humor, often self-deprecating, from their experience with the disease as means to neutralize potential stigma, provide a sense of power and protect oneself. Conversely, as means to protect oneself from being “judged” or “thought differently” about.

Nothing ever gets put in my way ‘cos I just knock it down and go for it anyway ‘cos I’m that type of person. Think you have to be with the condition you have to show it who’s boss [laughs] (F015)

I see it more positively I think knowing that although it may get the better of me sometimes I can do stuff and it won’t stop me, so yeah I think it has made me realise that I’m someone that has Crohn’s but that’s not the end of the world and you can still enjoy yourself even if times are hard sometimes (F023)

I guess because I have had it so long I maybe don’t find myself nerved by things anymore (R013)

#### Self-management

Many participants recognized diet as an important element of the controlling the disease and symptoms experienced, particularly through food avoidance. Participants reflected on using the internet or books to establish what foods they should avoid, however, soon after discovering that “one leaflet doesn’t fit all” and adopted a trial-and-error process. Despite identifying foods through this process, which often did not stop participants from consuming these and were “willing to suffer the consequences.” Managing stress was another self-management method, with many accomplishing this from resting, relaxation, walking, taking time off and not dwelling on things. Several participants mentioned incorporating Pilates, fishing, golf, and yoga into their lifestyle.

Short run its annoying having to restrict yourself [Diet]but a lot of people do it and it’s not the end of the world. Think I’d do pretty much anything or try anything really to help with my condition, just because I know how bad it can get (F023)

…you feel as though you can’t eat the things you enjoy and it, and it probably like sounds stupid but I love my Indians [laughs] erm and after 22 years I got fed up of following a diet I should eat and you, you know if it means spending the night on the toilet then I’ll probably do it (R032)

I try to do relaxation techniques, I feel as though my Crohn’s is very linked to my emotions (F009)

Making plans around the condition were commonly used to relieve stress, enhance control, and cope effectively with disease-specific challenges. Knowing where the toilet facilities were, carrying pain relief, being with supportive company who understood the condition and by avoiding certain foods/not eating if going to or at events were components of planning. Some participants expressed during times of stress or particularly during a flare up they preferred staying in their own home to avoid traveling, unknown environments and fear of lack of toilet facilities/urgency.

…there has been a few times I’ve had to come away from social things or if I’m out shopping by myself because I’m getting stressed with looking for where the toilets are… or I could have pain and I just need to get home, that’s where I feel most comfortable…if I do have an accident then I’m in my own home, it’s not embarrassing as that really is quite traumatic (R005)

…took me a few years for that but I’ll now happily say to my family and friends look I’m tired I can’t do today can we re-arrange and they understand (R031)

#### Social comparisons

Another coping strategy employed by participants was by positively comparing themselves to other adults with the same condition. Some participants compared themselves to those who they felt were more negatively impacted by the condition, seeing themselves as more “fortunate” or “a fraud” if their condition was not as bad. This was evident in those who had had surgery to alleviate symptoms or were not currently on any medications, conversely, those who had not had surgery felt they were “lucky” compared to those who did.

“I always thought I was quite lucky compared to some people with Crohn’s you know [pause 2 seconds] because I had surgery and haven’t had any problems since [pause 2 seconds] I almost feel like a fraud saying I have it because I feel so well after my surgery” (R030)

…so I’m fortunate in that aspect as the surgeries for Crohn’s aren’t er the best probably more life changing than the condition (R036)

I’ve never needed any hospital treatment or anything like that so I’m lucky in that respect [pause 4 seconds] I feel guilty to be honest saying I have the disease because I don’t experience the symptoms (R012)

#### Barriers to resilience

Some barriers to resilience were acknowledged. The invisibility of the disease and symptoms experienced, even during remission were recalled, in particular, the “you look fine” which created feelings of judgement and social anxiety. A few participants recalled comments made by members of the public when it came to using disabled toilets or needing to access facilities quickly. Leading to participants debating whether to disclose their condition and explain themselves.

You may look well sometimes but feel absolutely terrible on the inside so it massively affected what people thought of me (R015)

People always want to know your business, you get stared at if you even try to use a disabled toilet think that’s just British society for you (F015)

## Discussion

This study provides an insight into the complex health journey and challenges of people living with CD. Three overarching themes were revealed, along with 12 subthemes: (1) *Reactions* to presenting symptoms, emotions, and challenges at diagnosis; (2) *Reality* of living with the condition, seeking information, decision making, psychological challenges, experiencing symptoms/complications during remission and the impact on social life, education, employment, and relationships; (3) *Resilience* involving emotional adaptations, strategies on self-management, social comparisons as a means of coping and barriers to resilience.

During the pre-diagnostic stage, some individuals and primary care professionals viewed early symptoms as insignificant due to their mild nature, leading to participants to normalize and belittle their symptoms and explain them away with everyday events, a finding consistent with previous research.^17–19^ Not recognizing the importance or seriousness of symptoms and the challenges some faced when communicating with primary care professionals contributed to a delayed diagnosis, now recognized to have a detrimental impact on the treatment options available and increase the likelihood of hospitalization or surgery.^18,20,21^ With one in 10 people with IBD visiting a healthcare professional with symptoms 5 years prior to receiving a diagnosis,^22^ speeding up diagnostic investigations and early diagnosis is crucial. However, given that CD symptoms described are often also common symptoms of other digestive disorders, developing IBD-specific tools for primary care use may help with distinguishing the disease, regular monitoring, resulting in early diagnosis and thus improving health outcomes and reducing healthcare costs. In addition, primary care professionals should be especially mindful of anyone presenting with IBD-like symptoms and resort to existing training resources, a toolkit in line with NICE guidelines for diagnosing IBD, as developed by the Royal College of General Practitioners in collaboration with Crohn’s and Colitis UK.^23^ This educational resource may also be used to help support conversations, build the patient–clinician rapport, and take into account concerns, which a few participants felt were lacking.

A recent health campaign “Cut the Crap: Check for Crohn’s and Colitis” has acknowledged the need to raise awareness, encourage symptom checking and work with clinicians and policymakers to develop a patient-led national diagnostic pathway for individuals presenting lower gastrointestinal symptoms.^24^ These study findings demonstrate clear value in a proposed patient-led pathway and warrant enhanced diagnostic pathways and accelerated specialist referrals to ensure individuals are diagnosed and treated in a timely manner. There is also a need for patient experiences to be embedded in healthcare development to improve the quality of care and outcomes for patients.

Although an early diagnosis is crucial, so is the need for providing adequate disease information. The experiences of most in this study indicate that participants know little about CD, with many seeking research into the disease. Sometimes, this lack of awareness or misinterpretation of information caused fear, distress, and vulnerability in participants. For those recently diagnosed, these emotions were worsened if they required immediate surgery, hospitalization, or intravenous drugs or if they knew someone who had a bad experience with the condition. Many participants after receiving a diagnosis continued to seek information however in contrast to previous findings,^25^ exploring alternative therapies and surgical options was just as common as seeking dietary advice. It is also worth highlighting that a few participants also sought additional information on the hereditary nature of the disease and the impact the disease may have on starting a family, a topic not often discussed. Providing sufficient public education, information, and advice from healthcare professionals is warranted to avoid false perceptions or misinformation and may help individuals cope with the disease faster and better as it has in other health conditions.^26,27^

While information-seeking behavior has been seen to positively influence the process of coping and adjusting and improve self-management abilities,^28,29^ in the current study, it also negatively influenced surgical decision making, in particular stoma formation. Encountering conflicting or perceived negative information can exacerbate and lead to uninformed decision making. The National Institute for Health and Care Excellence (NICE) emphasize the importance of providing clear and balanced information when considering surgery,^30^ requiring early-stage discussions and multi-disciplinary team input.^31^ However, no high-quality studies have assessed the desired content of information that could provide barriers to base discussions and recommendations. In addition, there is also a need to explore when this information is provided, despite all participants agreeing it was the right decision many saw having a stoma as a last resort, driven by disease activity or failure of other treatments, and not as an option. Therefore, it is vital that reputable medical websites, expert opinions, and anecdotes are provided to participants to facilitate trustworthy information.

Discussions around mental and emotional health dominated conversations throughout. Sewitch et al.^32^and Mikocka-Walus et al.^33^ describe a series of psychological adaptive steps that occur after receiving a diagnosis; initial evaluation of disease impact, emotional reactions (distress, grief, guilt), behavioral response, seeking social support, modification of diet and various degrees of denial and/or disease acceptance, which were evident throughout. Many continue to experience an emotional roller coaster between these steps, often influenced by environmental and societal factors. Previous studies^2,4,34^ have highlighted similar influencing factors including fears of incontinence, avoidance behavior, lack of control, lack of understanding, coupled with the invisibility of symptoms and disease complications. All of which have detrimental psychological consequences leading to low levels of self-worth,^35^ identity loss,^36^ and low self-esteem.^37^ Our study also highlights the impact medication side effects can have on psychological well-being. For instance, social isolation as a means to control or protect oneself was evident throughout, primarily when the disease was active. Psychological distress due to body image changes should not be overlooked, it is imperative to understand what is important, be empathetic and supportive to the individual and ensure that the information on medication side effects is discussed to avoid further disruption.

Social isolation and withdrawal from relationships are common consequences in chronic illness;^38–41^ however, perhaps more so in individuals with CD due to a taboo around discussing bowel symptoms. This may explain the secrecy of some not wanting to disclose their diagnosis. Another reason could be due to fear of stigma, particularly in the workplace. Although some participants in the current study described employers as being accommodating, others experienced difficulties that led some to seek early retirement or self-employment status to control repercussions or the explanation of absences. A recent European survey of 202 IBD working-age participants, identified nearly a quarter of participants were on sick leave (*n* = 31) and another quarter were receiving a disability pension (*n* = 46).^42^ Being in receipt of disability pension, unemployment, and sick leave along with worries over job security and financial repercussions all further negatively impact psychological wellbeing.^43–45^ There is a need to increase awareness of the condition with employers to enable them to provide a supportive, accessible, and adaptative environment so as to not to make individuals feel pushed into taking a disability pension, choosing not to work, or opting for self-employment when they feel these are their only viable options.

Our study not only highlighted the detrimental impact the disease can have on employment but also on the martial relationship,^46,47^ friendships,^48,49^ and educational outcomes^50–52^ due to a lack of understanding, support, knowledge, or empathy from others. Therefore, perhaps not surprising that those with the condition limit their social activities, actively choose to stop seeking new relationships and lower their career expectations.^34,53^ Other means of managing the disease included adapting their environment to buffer the negative impact of stress, for example, avoiding unknown places, being with the supportive company, knowing where the toilets are, avoiding public transport, staying at home, and making plans around the condition. Pilates, yoga, relaxation, walking, and taking time off were also discussed as ways to reduce stress. Another interesting finding was food avoidance, while common in IBD^4,54–56^ to avoid certain foods that cause negative symptoms, some participants discussed not eating at all when going out or attending events. These beliefs and behaviors increase the risk of disordered eating or an eating disorder, incidences that are increased in CD.^56–59^ With up to 85% of adults with IBD experiencing malnutrition,^60^ individuals should be encouraged to speak with their gastroenterologist or dietitian to learn about adjusting their diet safely to meet nutritional requirements.

The burden of trying to live with unpredictability of symptoms and relapse was a constant challenge in an individual’s ability to be resilient. Participants described a variety of coping strategies to maintain resilience such as appreciating what they have, adapting to lifestyle changes, learning about the condition, being aware of limitations, and overall disease acceptance as means to neutralize potential stigma, provide a sense of power, and protect oneself. One of these methods notably included self-deprecating humor. This “fight or flight” response^61^ to a perceived threat or judgment was common, and while protecting and defending oneself is important, it is also important that challenges and experiences are not undermined but faced with acceptance instead of self-deprecating humor. Another interesting coping method was disease comparison, by relating their own experience to others often perceived as worse off. Unlike other chronic conditions^62,63^ where these comparisons were evaluated positively, in the current study, some of these comparisons were viewed negatively leading to feelings of guilt or feeling like a fraud. Imposter syndrome has been well documented; however, the impact of imposter syndrome on those with chronic illness remains sparse despite its strong negative impact on anxiety and depression,^64^ which those with IBD are more prone to.^65,66^ Understanding why this phenomenon occurs and ways of managing these challenges are vital to ensure the individual recognizes their own self-worth, reduces self-doubt, seek healthcare advice when required, and reduces the impact on mental health.

### Strengths and Limitations

A strength of this study was the trustworthiness of the data. Two independent coders of different backgrounds (exercise physiology and physiotherapy) conducted data analysis, enhancing the credibility of the research but also allowing different perspectives, open reflection, and discussion during theme development. Debrief meetings with the wider team with different backgrounds (research, clinical, and subject expertise) allowed for further reflection and theme development. The use of framework analysis allowed for comparability enabling the facilitation of patterns and differences. Another strength was the inclusion of a large sample size, which enabled a range of views of people’s journey at different stages of their health journey from initial diagnosis through to managing their condition to be collected.

An important limitation of the study was the inclusion of only participants with an inactive to mildly active disease, who were recruited from one hospital site, and of which all were of white ethnicity. Thereby affecting the social and cultural representativeness of our findings to a broader population. Another limitation was the inclusion of participants who have been diagnosed with CD for a long time, these participants may have experienced different diagnosis pathways, primary consulting, and overall disease management to those more newly diagnosed. Finally, participants had a prior relationship with the interviewer who completed study assessments and delivered the RCT. Therefore, it is possible that this rapport may have influenced the responses given.

## Conclusion

This study highlights the complex health journey and challenges faced by people living with CD on various of life and recognizes the need for coping strategies, resilience, and access to several support resources. Most importantly, these findings highlighted the unmet emotional needs and the emotional turmoil experienced by individuals at certain times throughout their diagnosis. We emphasize the need for targeted interventions, including approaches to address stigma and resilience to enhance psychological well-being at these stages and greater attention around psychological health as much as physical aspects of the disease. Overall, information from this study provides health care professionals with a greater insight into the psychological challenges and emotional complexities of the condition to facilitate a more holistic approach to planning care.

## Supplementary Material

otaf003_suppl_Supplementary_Material

## Data Availability

All data are available from the corresponding author upon reasonable request.
