# Supplementary material for: Reactions, Reality, and Resilience in Adults with Crohn’s Disease: A Qualitative Study
Source: Crohns Colitis 360. 2025 Jan 16;7(1):otaf003. doi: 10.1093/crocol/otaf003 (PMC11799741; doi:10.1093/crocol/otaf003)
Supplement: otaf003_suppl_Supplementary_Material [file otaf003_suppl_supplementary_material.docx]

**Supplementary Material**

1. **Interview Topic Guide** *(italicised sections denote where most data were collected for these findings)*

**Introduction (All participants n=41)**

Before we begin, our discussion will be recorded as part of the research process and transcribed for analysis. Your personal details and everything you say will be anonymised and kept confidential so it cannot be identified. We will talk about your experiences and views of taking part in this study, are you happy to go ahead?

**Disease History (All participants n=41)**

*Before we start talking about your experience participating in the research study, could you tell me a bit about your Crohn’s disease? From when you were diagnosed up until taking part in the research?*

- *What were your presenting symptoms?*
- *When were you diagnosed? How long ago? How were you diagnosed?*
- *What is your history with Crohn’s disease? Flare ups? Problems related to your condition? Problems experienced because of condition?*
- *What medications have you tried? How have you found them?*
- *Any surgical procedures?*
- *Have you tried anything yourself to manage your Crohn’s disease? Has this method been successful? How?*
- *Do you experience abdominal pain or joint pain as a result of your condition? How long have you experienced this pain? Is there anything you do to reduce your pain? Is there anything you find makes it worse?*

**Research Questions (All participants n=41)**

The next few questions will be focused on your experience participating in the study. *What were your thoughts when you heard about the research opportunity?* How did you hear about the research?

- From the information you received which of the following was most important and relevant when deciding to participate: 1. Invitation letter; 2. Participant information sheet; 3. Organisation (location/ timing of assessments)
- Was there any additional information you would have liked to receive that might have been helpful when you were deciding to participate?
- *Why did you choose to participate in the research study?*
- How did you find the study assessment at the hospital?
- *What did you think about the setting at the hospital?*
- How did you find the travel and length of the sessions?
- *What was your relationship like with the research nurse and clinical investigators?*
- *How did you find the clinical assessments carried out at the hospital, such as the stool and blood samples and physical examination?*
- How did you find the study assessments at the university?
- What did you think about the setting at the university?
- How did you find the travel and length of the sessions?
- What was your relationship like with the researcher and other investigators?
- How did you find the clinical assessments carried out at the university such as the bone mineral density scan, muscular performance tests and questionnaires

**Intervention Experience (Exercise Group Only n=21)**

*The next few questions are going to involve your experience in the group you were placed in. What was your initial preference, control or exercise? Why was this you preference?*

- *What did you think when you were allocated to the exercise group?*
- What did you expect from the exercise programme before you began?
- Could you tell me what you thought about the design of the exercise programme?
- How did you feel about completing three sessions a week? (too often, just right, not enough)
- How did you find the intensity of the programme? (too often, just right, not enough)
- What did you think about the length of the session? (too often, just right, not enough)
- What are your thoughts regarding the length of the programme? (too often, just right, not enough)
- What are your thoughts on the type of exercise used? (too often, just right, not enough)
- What are your thoughts about the setting of the exercise programme? Was it appropriate? Could it have been better? If so, how?
- *Was there anything that made it hard for you’re to complete the exercise programme?*
- *What made you keep attending the sessions?*
- Could you recommend are changes to the exercise programme if the study were to run again?
- *In regards to the researcher delivering the exercise programme, can you tell me what your relationship was like? How did this relationship compare with your relationship with other healthcare professionals?*
- Is there any aspect which could have been better? Do you think that the right sort of person delivered the intervention?

**Outcomes (Exercise Group Only n=21)**

- Has the exercise intervention had any positive or negative changes on you, such as:
- *Physical changes, anything you have noticed as a result of the exercise?*
- *Apart physical changes, how do you feel in yourself since completing the exercise programme?*
- *Do you see any changes in your condition after completing the exercise programme?*
- *Has it affected how you will manage you condition in the future?*
- *Has it changed your understanding of your condition? How?*
- *Do you see you condition differently than before?*
- Abdominal pain, has the intervention affected the amount or severity of the abdominal pain you experienced before commencing the exercise programme?
- Joint pain, has the intervention affected the amount or severity of the abdominal pain you experienced before commencing the exercise programme?

**Acceptability (Exercise Group Only n=21)**

The last few questions will focus on your thoughts of the acceptability of the exercise programme.

- How acceptable do you think the exercise programme was?
- *Would you recommend this sort of exercise programme to other people with Crohn’s disease? Why?*
- *Can you think of any reasons people with Crohn’s disease may not want to participate in this type of exercise? Or research study?*
- Do you think this type of exercise training should be offered on the NHS for people with Crohn’s? If no, why not? If yes, what would the exercise training look like, what would you like to see?
- If exercise training wasn’t available on the NHS, would you be willing to pay for it and if so much would you be willing to pay?

**End (All participants n=41)**

I think we have covered all necessary topics, is there anything you would like to discuss or add? Or do you have any questions?

- (End call) Thank you very much for your time its greatly appreciated.

1. **Individual Participant Characteristics**

| **Participant ID** | **Gender** | **Age** | **Employment Status** | **Age at Diagnosis** | **Duration of Diagnosis** *Years (Months)* | **CDAI Status** |
| --- | --- | --- | --- | --- | --- | --- |
| F001 | Male | 43 | Employed Full Time | 27 | 17 (216) | Inactive |
| R002 | Female | 36 | Employed Full Time | 31 | 5 (66) | Inactive |
| R005 | Female | 60 | Self-Employed | 30 | 30 (388) | Inactive |
| R008 | Female | 71 | Retired | 31 | 40 (484) | Inactive |
| R009 | Female | 48 | Employed Full Time | 17 | 31 (383) | Mildly Active |
| R012 | Female | 47 | Self-Employed | 37 | 10 (142) | Mildly Active |
| R013 | Female | 27 | Employed Full Time | 11 | 16 (192) | Inactive |
| R014 | Female | 46 | Employed Full Time | 43 | 3 (36) | Inactive |
| R015 | Female | 63 | Retired | 37 | 26 (312) | Inactive |
| R016 | Female | 55 | Employed Full Time | 36 | 19 (228) | Inactive |
| R017 | Female | 25 | Employed Full Time | 23 | 2 (24) | Inactive |
| R018 | Male | 37 | Employed Full Time | 21 | 16 (192) | Inactive |
| R021 | Female | 60 | Unemployed | 23 | 37 (444) | Mildly Active |
| F002 | Female | 33 | Employed Part Time | 31 | 2 (18) | Inactive |
| F007 | Female | 43 | Employed Part Time | 38 | 5 (60) | Mildly Active |
| F008 | Male | 38 | Employed Full Time | 33 | 5 (60) | Inactive |
| F009 | Female | 59 | Retired | 18 | 41 (492) | Mildly Active |
| R024 | Male | 38 | Unemployed | 22 | 16 (192) | Mildly Active |
| F015 | Male | 49 | Self-Employed | 32 | 17 (204) | Inactive |
| R027 | Female | 44 | Self-Employed | 25 | 19 (228) | Inactive |
| R028 | Female | 51 | Retired | 22 | 29 (348) | Inactive |
| R030 | Male | 50 | Employed Full Time | 46 | 4 (48) | Inactive |
| R031 | Female | 56 | Employed Part Time | 18 | 38 (456) | Mildly Active |
| R032 | Male | 54 | Self-Employed | 33 | 21 (252) | Inactive |
| R033 | Female | 60 | Retired | 50 | 10 (120) | Inactive |
| R034 | Female | 36 | Employed Full Time | 31 | 5 (60) | Mildly Active |
| R036 | Male | 59 | Self-Employed | 51 | 8 (96) | Inactive |
| R037 | Female | 70 | Employed Part Time | 30 | 40 (484) | Inactive |
| R038 | Female | 37 | Unemployed | 32 | 5 (60) | Inactive |
| R039 | Female | 38 | Employed Full Time | 20 | 18 (216) | Mildly Active |
| F018 | Female | 52 | Employed Part Time | 20 | 18 (216) | Inactive |
| F019 | Female | 27 | Employed Full Time | 26 | 1 (12) | Inactive |
| F020 | Male | 67 | Retired | 12 | 55 (660) | Mildly Active |
| F021 | Male | 49 | Employed Full Time | 31 | 18 (216) | Inactive |
| F022 | Male | 51 | Employed Full Time | 42 | 9 (108) | Inactive |
| R041 | Female | 47 | Employed Full Time | 44 | 3 (36) | Inactive |
| R044 | Male | 66 | Employed Full Time | 56 | 10 (120) | Mildly Active |
| R045 | Female | 51 | Unemployed | 33 | 18 (216) | Inactive |
| R046 | Female | 68 | Retired | 34 | 34 (408) | Inactive |
| R048 | Male | 58 | Retired | 28 | 30 (388) | Mildly Active |
| F023 | Female | 46 | Employed Full Time | 43 | 3 (36) | Mildly Active |
| CDAI, Crohn’s Disease Activity Index | | | | | | |

1. **Additional Participant Quotes**

| **Theme** | **ID** | **Quote** |
| --- | --- | --- |
| Reactions | F001 | I ended up going private because I didn’t feel like I was been listening to by my GP |
|  |  | I’ve had some awful experiences of abdominal pain, that will always be with me like burned in my head. |
|  | R002 | I went to the doctor and he just said oh its just irritable bowel syndrome so that went on for about a year |
|  |  | At the time I was really overwhelmed with everything [pause 4 seconds] I just had my daughter I was ill with something that I’d never even heard of. It was really hard trying to adjust with a new born and not being able to lift her or take her out because of the Crohn’s and then obviously found out it was a fistula caused |
|  |  | I was cramping up I would be sick it was like I no control over my body at all….just had to watch what I ate all the time |
|  | R005 | My intestines had got to such a state they couldn’t repair |
|  |  | I had to continue drinking some some kind of [pause 2 seconds] erm supplement until I could get to a weight to have an operation and then erm [pause 2 seconds] that’s when they did a resection. |
|  |  | I had to go to hospital to be tube fed for about erm well until I got to a certain weight |
|  | R008 | I had constant diarrhoea and sickness and, and it went on like that for a while and I lost a lot of weight and the doctors didn’t think it was anything and then I had my son we he was born, the Crohn’s whatever it was seemed to settle down but then it started up again when he was about 6 months old and I used to vomit for three days then have three days of not vomiting [pause 2 seconds] I lost weight I went down to 5 stone and the doctor, when I used to go down to the doctors they used to say things like how’s your marriage, I see you’re selling your house you know how’s your relationship and they, I used to start to go down every week because I thought either I’m ill or I’m going crazy |
|  |  | I was quite ill and they said they had to operate straight away and I had some bowel removed |
|  |  | I must have been late 20’s early 30’s by the time they actually diagnosed me but I was getting symptoms before that for a good while, maybe 2 years before I got diagnosed with Crohn’s just I wasn’t listened to because they just thought I was losing weight because I was married or had children |
|  |  | My own doctor put his head down as I think they thought I was a hypercondriact as well you know, then they didn’t take women seriously [pause 2 seconds] I went for years experiencing vomiting and pain, oh pain like you wouldn’t believe and nothing just that I must be overreacting as it can’t have been that bad [pause 2 seconds] you know I was almost pleased to be diagnosed with Crohn’s just to show the doctor I wasn’t making it up |
|  | R009 | I was given about 4 hours to live and so they rushed us into surgery and took out large bowel and did a join em and then about a week later I found that the join didn’t work and then gave us an ileostomy at that age that was 19 And it was sent off for pathology to London and it came back as Crohn’s. That’s the history of my Crohn’s really it’s sort of like crazy emergency and then nothing |
|  |  | It just came out of nowhere, it, it was terrifying to be honest because, as I said back then you just didn’t know what things were |
|  |  |  |
|  | R012 | I’d had food poisoning, erm I’d had campylobacter and erm ever since that point my bowels had never been 100% right, just been a change in consistency, frequency and all of that [pause 3 seconds] originally they thought it was IBS |
|  |  | I’m a bit nervous now going to hospital, I don’t want to go in and come out with a stoma or have had any other surgery. The longer I have the disease the more chance that can happen and I don’t think it’s something I prepared for if I’m honest |
|  |  | It was a bit of a shock being diagnosed, I’d never heard of it before so it was quite a lot to take in and consider how it was going to effect my job, my life, starting a family erm [pause 3 seconds] I remember having a lot of questions, being told what the disease was and just not taking any of it in |
|  |  | I’m a bit nervous now going to hospital, I don’t want to go in and come out with a stoma or have had any other surgery. The longer I have the disease the more chance that can happen and I don’t think it’s something I prepared for if I’m honest |
|  | R013 | When I was 11 my mum took me to hospital because I had diarrohea and vomiting and I think there was blood in my stool [pause 2 seconds] erm and they couldn’t figure out what was going on so I stayed in there about 4 weeks and then they found out I had Crohn’s |
|  |  | I think it started first with a swollen lip when I was 8, and they took a lip biopsy and they did allergy test and that didn’t show anything. But they think now that it was a presign of the Crohns two years down the line. But I think because I was so young I didn’t really know what was going on |
|  | R014 | I was diagnosed in 2016 erm the symptoms was erm [pause 2 seconds] I had a fissure and I went along with that as it wasn’t healing up |
|  | R015 | GP just advised me to shut up and put up |
|  |  | I think I’d always had like what I’d class as stomach problems from about the age of 12 |
|  |  | I mean I’ve had Dr’s over the years and some of them have been awful not believing what you are saying and just not listening that’s what em i, I just get angry |
|  |  | I just wasn’t being respected by the people [GP] who were treating me |
|  | R016 | I was pregnant and I was getting really really severe pains [pause 3 seconds] I was told it was probably pregnancy pains. But then I was really poorly towards the end of my pregnancy which was really scary as I thought I was loosing the baby [pause 2 seconds] but I ended up being admitted straight away and having part of my bowel removed. |
|  |  | I had a big mass and because I was pregnant they couldn’t really do scans or anything so they were thinking it was maybe appendicitis but when they went through key hole surgery they realised I had Crohns |
|  |  | It was more of the fact I was about to have a child and I was this ill and thinking I couldn’t manage, was I going to die, like id only heard of it from a friend who was really ill from it and I was just in a right mess thinking all sorts |
|  | R017 | I was 23 it started kind of with tummy cramp erm and then erm and then rectal bleeding and erm I started to loose weight very quickly. I think I had symptoms long before I was diagnosed but I just thought it was period cramp |
|  |  | It can be quite scary having blood in your stool I was only around 22, 23 and was away from home for uni and I just remember having that phone call with my mum erm and coming home from uni because I was scared |
|  |  | Things started to get a bit worse I ended up getting abdominal pain and just feeling really ill [Pause 2 seconds] and then obviously I went to my GP, oh and there was blood as well in the stools which I hadn’t had before |
|  |  | I do think when your being diagnosed the last thing you do is ask questions about why they are doing scans or what they are looking for [pause 3 seconds] erm because your so ill and so tired of feeling that way that you just sort of accept what it is they are doing |
|  | R018 | I presented as an emergency admission in 2002 so I was at uni at the time and Id just finished my last uni year and hadn’t been feeling well for quite sometime and had some weight loss that came quite gradually so I didn’t really recognise it it was I felt awful, I felt awful for a long time erm and then I went well I bounced back to the family doctors abit and they didn’t really know what to do and then I think one of the GPs got a blood result back that was way out so they said okay go to emergency admissions to find out what was going on. And I didn’t leave hospital from that time, they ended up doing an emergency resection and recovery kind of took place from that time |
|  |  | I went down for the emergency resection they didn’t know what it was erm you know they give you the talk about preparing you for cancer or things like that which was quite scary |
|  |  | With all these new thoughts put in my head I just kept thinking, like, whats going to happen to me and erm you know you aren’t allowed people with you down there so I was just by myself in the bed it was pretty awful |
|  |  | In hindsight knowing what the problem was I could see symptoms going back about 5 years erm but I just adapted to it |
|  |  | I never really expected it to be Crohn’s I thought it was going to be IBS or an intolerance or something because I’d noticed certain foods starting to not agree with me and causing abit of pain. So you do what any normal person would do and stop eating that food but when it becomes most things it got to the point where I didn’t want to eat anything because I knew it would cause me pain and problems later on [pause 4 seconds] but I only look at the now and realise that’s probably why I was loosing weight because I wasn’t eating much |
|  |  | I guess its just hard to know what was stress from uni and what was the Crohn’s plus there was always bugs going round so whether it was just a bug or what |
|  |  | I was relived it was Crohn’s because it wasn’t cancer [pause 2 seconds] but then I’d never heard of Crohn’s so didn’t know what was coming |
|  | R021 | I had to have a blood transfusion and er all my periods had stopped as well I was really poorly but it took a good few years before I even got a diagnosis |
|  |  | I did have them endo colonoscopy things and what have you. [Pause two seconds] I had loads of them erm and I had bowel washes and everything [paused 3 seconds] it was a lengthy process before I got told what it was and the tests and what have you aren’t nice at all there couldn’t be a blood test that tells you could there [laughs] |
|  |  | They first thought it was ulcerative colitis and that was 1981 [pause 2 seconds] and then erm [pause 2 seconds] my son was born 1991, maybe 93 94 that’s when I had to have my operation and that’s when it was [pause 1 second] That’s when it was Crohn’s disease so it was a while before I got a diagnosis and obviously had to go straight in and have surgery |
|  | F002 | I was going to the toilet a lot, which isn’t something you want to talk about |
|  | F007 | Like mentally which was made worse by the fact my GP just thought it was a bug and gave me these drinks |
|  |  | I couldn’t keep them down to be honest, so I didn’t take them I just knew something was wrong in myself and it was frustrating that I wasn’t been listened to. |
|  |  | For a good few months I went to the GPs and it was only when I lost a lot of weight did they refer me, erm [pause 2 seconds] to the [HOSPITAL NAME] and I end up having part of my bowel taken out [pause 3 seconds] so yeah it went from being told it was a bug to having surgery |
|  |  | My mum always said I was a fussy eater and didn’t tend to eat much and then obviously years later im having surgery and being diagnosed with Crohn’s |
|  |  | It felt like longer than a week waiting to find out what’s wrong with you and obviously you spend that time Googling things and god awful stuff comes up so I don’t know if I was pleased it was the Crohn’s |
|  | F008 | I think it was the not knowing and not being listened to but as well I had no energy to fight with the GP to be referred it was only when my mum decided to come with me and that’s when I got referred |
|  |  | Basically I never had any major issues and it was round about 2013 that erm it started for me and came completely out of the blue. I was off food feeling sickly but that was kind of pretty much it for me no diarrhoea or pain or anything like that just not really eating and feeling sick |
|  |  | A bit surprising considered I had no bowel changes I ended up having part of my bowel removed I never really understood how that could be the case |
|  |  | Due to the severity of it and er other kind of complications they said it was better to have the surgery so I just went straight in for that |
|  |  | My first sort of introduction, this is what you may have, this is what you’ve got we are going to take it away [laughs] not the best sort of thing as you’d like a bit more information on why you have it |
|  |  | Like why do some people need surgery and some people don’t yeah its still all unknown guess we are all still learning |
|  | F009 | And it wasn't until it was six months that they discovered I had Crohn's disease after that on occasions when I was ill. |
|  |  | I wasn’t getting any nutrition and starting to feel really sluggish |
|  | R024 | The truth, it was awful, the first oh year, first two years it was bad it was hell. |
|  |  | Had like a er think it’s a fistula and then from that they diagnosed me with Crohn’s |
|  |  | Abdominal pain I’ve had that since before I was diagnosed, I think its like everyone though there’s worse days and better days I think it just depends on what I’m doing, what I’m eating and how I feel yano in myself. Think stress doesn’t help it |
|  | F015 | they said basically came back with it was severe constipation and maybe a bit of IBS |
|  |  | I just basically coped and managed it [pause 2 seconds] I’d be, you know off school for a couple of days here and there until the symptoms disappeared erm and basically through me late teenage erm, me late teenage years I coped with it. |
|  |  | I just lost lots of weight |
|  | R027 | I also had an endoscopy I didn’t really know what was going on to be honest I’d never been to hospital before and then it just felt like id never left |
|  |  | After a few visits to the doctor I finally got one that erm said I had some sort of bowel disease |
|  | R030 | It was a gradual increase of me just generally not feeling well which now I know and realise but it just came out of nowhere [pause 2 seconds] and then the pain came and I knew something wasn’t right I just knew. I took myself into hospital, I was actually kept fed up of going to the doctors to ask for help as I don’t think quite believed it [pause 2 seconds] and that’s the worse thing about it |
|  |  | My GP was convinced I had a food bug poisoning and he kept giving me these I can’t even remember they were called, and I went back to see them as I was still in discomfort and he basically said it was impossible and I just said why would I lie about pain [pause 2 seconds]. I don’t want pain, but I’ve got pain and he didn’t believe me. That was the most upsetting thing about it. |
|  |  | I’d never heard of it you know before getting diagnosed [pause 2 seconds], I’d never had any problems before it, it just came out of the blue that was the frightening part about it |
|  | R031 | I went straight to the dr and again he just gave me some tablets. I think I’d tried every tablet known to man [laughs]. I just kept going back and going back and he just said I’ll have to admit you and the lady at the hospital at the [HOSPITAL NAME], that was about the July time or the August time as I was about to go on holiday and I asked the lady she said it won’t make whatever you’ve got any better or any worse but you may as well go on holiday, and when I was there I may not as well have been on holiday I basically couldn’t, I spent most the week in the hotel room [pause 3 seconds]. And when I came back that’s when I went for the endoscopy and that was basically it. |
|  |  | I think its [joint pain] more or less since I was diagnosed, it’s not, it’s not a huge huge factor but it is there. It doesn’t restrict me as such but it’s there and its uncomfortable |
|  |  | As a result of my Crohn’s disease I think because my immune system doesn’t quite work properly, erm I had well got an underactive thyroid, erm I had [pause 2 seconds] since I had me erm ileostomy I’ve had erm kidney stones because I apparently produce some something more than any other person and plus I think because of dehydration [pause 2 seconds], erm I’ve had my gall bladder removed as well and they reckon that was as a result of erm [pause 2 seconds] they described it as your gallbladder has this washing up liquid that washes the fat away but it wasn’t doing that, so erm because of that I had, I had that. I’ve had a few blockages, erm I think because my iron levels have been very low erm so its had a bit of a knock on effect [pause 3 seconds] erm, so yeah probably more problems than the actual sort of condition itself |
|  |  | They removed some of my bowel a few years before my ileostomy which helped a little bit at the time but didn’t improve things greatly so I knew I would need more surgery at some point so having the ileostomy wasn’t that much of a surprise |
|  | R032 | Very very bad cramps erm, there was blood when I used the toilet |
|  | R033 | I haven’t had any other problems than that its just been a chronic bowel which I may have contributed to something like irritable bowel syndrome had I not known otherwise |
|  | R034 | I just noticed when I used to get the bus to work that I was getting really tired [pause 3 seconds] but erm like physically and mentally I just got drained and then the symptoms started |
|  |  | Horrendous abdominal pain that I couldn’t move, it was there for hours and I went to A&E to see someone. |
|  | R036 | But I went to the GP and he thought it might be an appendicitis so he, so he rushed me off the RVI |
|  |  | I did have other stuff like bloods and a scan but everything came back clear so they didn’t know what was wrong until the camera test. And to be honest I think that was only done because I was er persistent [laughs]. But its easy to see how it er gets missed for so long, you hear, well people go for years sometimes without knowing they have it |
|  |  | I just remember getting these stomach cramps when I was doing work, you just put it down to stress, everyone gets a niggly tummy when they are stressed and working for myself its even more stressful because if something goes wrong you have to do with it. |
|  | R037 | Well from late teens, erm through to my twenties I did have [pause 2 seconds] really severe bouts of stomach pain and sickness and erm not feeling able to get out of bed and and erm things like that but I didn’t know what was the reason for that and I never went to a doctor |
|  |  | They decided to remove that portion of the bowel and that under scrutiny, I think it was the surgeon, that recognised the erm condition and asked me how long I’d been suffering from Crohn’s, I said I didn’t even know I had it, that was a huge shock |
|  |  | I was rushed into hospital for emergency surgery and it was at that point, that I was told I had Crohn’s disease |
|  |  | He thought it sounded like an appendicitis because I’d been vomiting |
|  | R038 | I think at the time I had loads of family, like issues going on and I just couldn’t deal with me being ill so I wished it would just go away really |
|  |  | They treated me for years for colitis and nothing seemed to work fully, I always felt erm well I knew myself something didn’t feel right, I wasn’t well [pause 4 seconds]. Then they did a scope and then said it was actually Crohn’s, but me on better medication and that, I’m under better control now |
|  | R039 | I just experienced an extreme amount of fatigue |
|  |  | At the beginning it was mainly stomach pain, but I didn’t know what it was I just thought it was because my period was due |
|  | F018 | I believe I had the camera up and down as well and that was when they said Crohn’s disease. It wasn’t very pleasant, it was actually one of the worst things I’ve ever had and they try and do them every few years and I just say no [pause 3 seconds] I just can’t go through it. I don’t mind, things like scans I can do but the cameras are the worst part of the disease |
|  |  | At the time I was really overwhelmed with everything [pause 4 seconds] I just had my daughter I was ill with something that I’d never even heard of. It was really hard trying to adjust with a new born and not being able to lift her or take her out because of the Crohn’s and then obviously found out it was a fistula |
|  | F019 | The pains in me stomach I would say a little bit before being diagnosed with the Crohn’s erm, ‘cos that’s one of the reasons why I went to the doctors like ‘cos how quick I needed the toilet erm and for the pain in me stomach it almost felt like a stitch feeling in the right hand side of me stomach. Erm, so that’s when I thought there’s something not right and that’s when I did go to the doctors and they just put it down to IBS |
|  | F020 | It took so much from me |
|  |  | The first symptoms I had were swelling of me lips and there was a lot of ulceration around my mouth that appeared, my consultant said it was the first Crohn’s he had seen that had appeared as it had |
|  |  | I think the symptoms presented themselves it must have been early 60s and then by the time they had actually worked out I had Crohn’s but they didn’t tell me that because I’d gone off to college after I’d had the skin tags removed. |
|  |  | It would be 5 or 6 years later when I suddenly started experiencing erm passing blood, feeling weak and I went back to the hospital and then they seemed to know that I had Crohn’s and that it had suddenly become particularly bad. And within a 12 month period I was admitted erm and had the first bit of bowel removed. |
|  | F021 | Abdominal pain started a few years before I was diagnosed with Crohn’s [pause 3 seconds] which just gotten worse over the years |
|  |  | It was about 4 years after I was married [laughs] so it was about 1998 so yeah, everyone always says it was married life that caused it [laughs] |
|  |  | Initially I was taken in querying an appendicitis because it was lower right side sort of abdominal pain |
|  |  | Not really heard a lot about Crohn’s it was a little bit of what is that sort of and how did it manifest itself I was then transferred to the [HOSPITAL NAME] where I had a resection because an abscess had formed as well, well they actually drained the abscess in [HOSPITAL NAME] and then I went across to [HOSPITAL NAME] for the surgery |
|  | F022 | I’d been having so many problems from probably about my early 20s but I never did anything about, well, sorry I did. I kept going to the doctors and they kept fobbing me off saying I’d got an irritable bowel erm. |
|  |  | How it was finally diagnosed was unfortunately my bowel, my large bowel had ended up getting infected ‘cos of the disease and the infection had caused pulmonary embolisms so I ended up getting clots on both my lungs that’s how the doctors described it. The infection in my bowel had caused the disease in the bowel area to clot, those clots had then transferred to the lungs so everyone was completely confused ‘cos I hadn’t presented with any DVTs or anything like that so as a result of that they removed a section of my bowel and then as a result of me continually saying something’s not right can you tell me what’s going on here they eventually referred me to a gastroenterologist who said, did his examination and said no, no, no, you’ve got Crohn’s |
|  |  | I mean my issue, my condition, the biggest thing for my condition for me is obviously having to go to the toilet very quickly and stuff like that |
|  | R041 | I was diagnosed with bleeding piles but then a few more trips to the doctors erm I seen a new doctor and then he said no, erm I think you might, I think you might have a fissure so I then went to see another doctor at the [HOSPITAL NAME] who did a, a erm [pause 2 seconds] like an exploratory examination [pause 2 seconds] and then I still wasn’t, I still wasn’t right I still had the fissure a few more trips to the doctors and then they decided to send is to a gastro doctor so that’s when I seen my current doctor and I wasn’t diagnosed until I got a colonoscopy and and endoscopy [pause 4 seconds] and that was how I was diagnosed so go there eventually [laughs] |
|  |  | I hear people go for years without being diagnosed but I just don’t know how you can put up with those symptoms for so long [pause 3 seconds] I was ill, really ill |
|  |  | Being recently diagnosed I think that’s what I found most difficult because it just came from nowhere erm, no one in my family has it so why do i? |
|  | R044 | Yeah I think it was difficult erm going so long without knowing what was wrong and them thinking it was a range of other issues was really tough, felt like it dragged [pause 3 seconds] it was the longest and one of the toughest times I’ve gone through, when they diagnosed me I didn’t know whether to celebrate with joy it wasn’t the other conditions they thought it was or to cry [pause 4 seconds] but it’s one of those things isn’t it |
|  |  | I was on holiday and I got off the plane and I ended up in hospital in Cyprus for about 4 days and they said it was gallbladder, so once I got back they took my gallbladder out. I think that was very similar to what I experienced later on because I got admitted to hospital and they were going to remove my appendix and that was the start of it of being diagnosed with Crohn’s. I don’t really know if my gallbladder was a problem or whether it was the Crohn’s but at least I got a diagnosis, just er took a while to get to the route of the problem and to start treatment. |
|  | R045 | I actually went to A&E and I got admitted twice from the pain I had [pause 5 seconds] paracetamol just didn’t touch it, I mean I ended up having morphine and even then it was still there not as bad but still a niggle erm. It’s strange, sometimes it can be quite a sharp pain that’s quite brief and then other times it’s continuous it just doesn’t give you a break [pause 5 seconds] those were the times when I went to hospital. |
|  |  | I think you get so used to the pain being there, it would be there and id just think that was normal because that’s how it was |
|  |  | It was at the hospital they started all the horrible, all the horrible meals that you eat, the x-rays and the scans [pause 3 seconds] not a great experience of the condition [pause 2 seconds] like why couldn’t it have been anything else but bowel they shove cameras and tubes everywhere [laughs] |
|  | R046 | I went to the doctors, and they just kept saying come back when the pain is there, which was difficult because it was intermittent. But I mean we are talking years ago and things were a lot different back then, not much of the condition was really known you know not like now |
|  |  | They said straight away that I’d need to have surgery for it because it was so bad, and if I didn’t have it then it would definitely be in the next few years so that was a shock to the system |
|  | R048 | A junior doctor stepped out that I had been misdiagnosed with Colitis and no one had actually told me that it was Crohn’s. |
|  |  | Abdominal pain definitely was a factor in me knowing there was something wrong |
|  |  | It’s hard to know what is the Crohn’s and what is a bug or food poisoning so it is hard, you just get so used to having it you get used to the symptoms and feeling a certain way. |
|  | F023 | I had to get, like a hysterectomy and while were in the found endometriosis and sort all that out, so it was like really sore afterwards and I just kept going back to the doctors to say it was like still sore and still there after having the surgery. But they just kept telling me it was early days, it was always because I’d just had surgery and it was a big surgery. And it was like months and months and months afterwards and then I eventually got listened to |
| Reality | F001 | You know, that's just the way it is, the more fatty sort of foods and the one everyone loves don’t sit very well. I think its all lifestyle as well you know, id rather carry on eating healthy. |
|  |  | I've been married twice and I've got a quite a stressful job, a lot of responsibilities, get that kind of thing and get on top of, you know, and try to switch off. |
|  |  | The effects of the medications were horrible and made me feel so bad and it starting effecting my liver so I got moved on to the infliximab and that was the change, the biggest change that’s just given me a better lifestyle and management of it |
|  |  | Maybe a hot water bottle on my stomach. Just something other than trying to sit and wait until it went because that would just be torture |
|  | R002 | Probably sitting down for long periods of time doesn’t help my joints or the abdominal pain really so moving around and being active is helpful |
|  |  | Occasionally I do get a bit of pain where my scar is but apart from that nothing |
|  | R005 | I do experience abdominal yeah, erm since I was diagnosed [pause 2 seconds] it feels very crampy but its never got as bad as the first time as I had it, but I was on a vegetarian diet then so I don’t know if it was to do with that whether that’s why it was so painful |
|  |  | All these joint problems which seem to go with it to. |
|  |  | I wouldn’t get it all the time its just when I have flare up’s really I mean I was pregnant [pause 2 seconds], I had two more children after I was diagnosed, both times I was fine so I did contemplate having lots of kids [laughs] as that keeps it at bay [laughs]. |
|  |  | I’ve tried azathioprine and that made my quite headachy and sick and that didn’t seem to do very much |
|  |  | I’ve had some fistulas one in my bladder the first time, then another one going down from [pause 2 seconds] I get very confused with this it kind of [pause 2 seconds] went vertically down through my intestine so they took quite a bit out I’m not sure [pause 2 seconds]. I’m not very good at remembering what they did. I couldn’t tell you fully what they did they use to many technical terms for me I just trust what they do and thankfully they have been great [laughs] |
|  | R008 | I also get that skin thing mainly on my arms and nothing seems to get rid of it [pause 3 seconds] it’s a nightmare during the summer as I don’t want to wear short sleeve clothing because I don’t want people to see it and ask questions |
|  |  | Oh god forbid my children or grandchildren get it it just takes over your life [pause 2 seconds] you know my life revolved around being ill and looking after my children, it definitely affected me being able to work |
|  |  | I think maybe that’s a problem though as a lot of people put up with the pain because its not as bad as perhaps it was before so they don’t think anything of it but you know that’s not good. But I don’t want to bother the doctors with something that may not even be because of the disease [pause 3 seconds] its difficult to know what is best to do |
|  |  | I take paracetamol or I have a warm bath you know things like that or I go to the toilet and try to relive my bowels that always seems to help |
|  |  | You don’t always feel comfortable going to the bathroom especially if its in public or at a friends house, oh no, I just wait until I get home |
|  |  | I haven’t told my friends, it’s not something that I’ve particularly wanted to share and I’ve been well so there’s been no need to tell them you know |
|  |  | When they mentioned having a stoma, I was very against it, all you hear are stories and they are smelly |
|  | R009 | Abdominal pain erm, I think is definitely what I eat and just you know, just make sure you eat the right kind of things and catching bugs I think is more painful and you’ve also got a smaller system so you feel worse. I think with joint pain what makes it worse is damp weather, similar symptoms, erm the same for everybody who’s got arthritis, damp weather, if me magnesium is rubbish |
|  |  | You have residual problems like joint aching, rubbish skin, dry eyes and I don’t think it’s until somebody tells you it’s part of the condition that you actually know |
|  |  | Overdoing it I just think is sometimes with Crohn’s and I’ve learnt with fatigue to do with just general stuff, you know, is you have to take time to rest and you know what hours I work, bonkers, so erm, and stuff like that you want to be as normal as possible but now and again you just have to say right it’s OK to sit and watch 3 hours rubbish telly |
|  |  | Rest, lots of it and having a support my family are great and so supportive when I’m going through a rough time |
|  |  | It was the aching joints which just used to throb and that’s why I had to give up my job, erm, I gave it up because as a spa therapist you use your hands to do massages and facials, the pain just became unbearable |
|  |  | Some days are good others not so good but you get on and can only do what you can [laughs] actually I say that, but I’ll push myself, I work now and travel a lot for my job and it takes it out of me completely but I’m getting somewhere I haven’t in years and I don’t want my boss to think I can’t do. Do you know what I mean? So I just get try and get on with things like if it was urgency or pain It would be more of a problem but with fatigue you just have to push yourself through |
|  |  | I think just having some time to let my body do what its doing, you can’t always know when your gonna get it [abdominal pain] which is what is annoying er supermarket in the dentist it can be a pain and get in the way but usually, my friends know when I get the pain as I just go quite and then sometimes leave if need to |
|  |  | Joint pain, I would say is a mixture of a knackered body at 50. The Crohn’s I’d also, is erm, oh what I did have for years and years was a magnesium deficiency and that can sort of cause you sort of joint and muscle pain, erm, and I get really jumpy legs on a night time so I have a couple of amitriptyline for that because, but I think is, you know, How much of that is Crohn’s and how much is general life old age and probably a history of, well there’s a history of arthritis in the family |
|  | R012 | I haven’t had any surgery, I’m fortunate in that aspect definitely as when I was diagnosed I did do what your not meant to do and look it up online and the surgical stuff really frightened me and I am very against having anything done |
|  |  | Certainly abdominal pain erm, definitely erm, I would say probably most days a little bit and obviously working up to bad days where it’s just unremitting really, the cramping and the pain and the bloating |
|  |  | There’s not much you can do that’s what I’ve found any way [laughs] other than naturally working it’s way out you know [pause 2 seconds] I think sometimes though its hard to know when the pain is not right or too much that you should get checked out [pause 2 seconds] because the pain is just there |
|  |  | It’s difficult having the condition and juggling two children |
|  |  | To be honest some of those foods probably didn’t agree with me prior to the Crohn’s, there’s probably in my family a slight history of a sensitive bowel [pause 2 seconds] I mean things like particularly garlic I can’t really take, onions, spicy foods erm you know and not too much fruit either I always find are uncomfortable [pause 3 seconds] |
|  |  | I get a lot of support from my husband [pause 3 seconds] he’s been great over the years and I really don’t know what I would have done without the emotional and mental support |
|  | R013 | Maybe certain foods I find cheese makes the stomach cramps worse, and stress with work [pause 2 seconds] the colder weather and doing too much always makes my knee hurt more |
|  |  | I love a hot water bottle [laughs] I do find that helps both the stomach cramps and joint pain |
|  |  | With the pain before my stoma it was just like grippling, like its hard to describe it just felt like someone was stabbing me really [pause 3 seconds] whereas like after my stoma it was more of a twinge but manageable like it was uncomfortable |
|  |  | That made me put on weight and then although I was well or like better I didn’t feel good in myself because I’d put on weight and had a moon face off the steroids [pause 2 seconds] so most the photos I have are of me with a lot of weight on or with the steroids moon face which isn’t very attractive |
|  |  | I guess because I knew the medication made me feel sick and like drowsy and then obviously changing my appearance which is hard anyway going through your teens |
|  |  | When I was 18 and I seemed to be fine and it was quite controlled up until then, but then I started to get quite swollen joints like my left knee was really bad I couldn’t walk |
|  |  | It was nice to see how there for me my friends were [pause 2 seconds] we went for a walk up this mountain which obviously didn’t have toilets and my bag burst, obviously you have no control over it, so like it burst [pause 2 seconds] I was like oh my god what do I do im on a mountain and my friends huddled round me to cover me while I was able to change the bag [pause 4 seconds] so yeah it’s things like that you just have to deal with but its nice to know my friends are there for me |
|  |  | I started steroids at so young and they did give me these tablets to take with them, but I just didn’t because I didn’t like them and didn’t really realise what they were meant for so like I didn’t take them which is probably my own fault why I have problems with my bones and joints |
|  | R014 | I do have quite a few flare-ups, erm [pause 3 seconds] at the moment the ones I’ve been getting they said have been due to stress |
|  |  | I work full time Monday- Saturday and often do shifts on a Sunday so sometimes its hard erm to tell the difference between being tired from work or feeling fatigued |
|  |  | I was never advised to try anything else apart from just stick to the medication just erm just in case it irritated it so I haven’t done anything because I’m not really sure what I can do |
|  |  | It’s overwhelming, you sleep and that doesn’t help, you wake up after 8 hours sleep and could sleep for longer |
|  | R015 | It was very distressing to live with, completely affected me personality and I think that just reaffirmed even more that I had Crohn’s disease |
|  |  | I had to have it done again [surgery] and in the end I’ve just had to give up. It was a very low point in my life I think I just had as I mentioned before I lost who I was I just gave up |
|  |  | I’ve always had like an upset stomach and a lot of mucus, all that type of thing, all the unpleasant side effects |
|  |  | The abdominal pain that’s been mainly after I’ve been diagnosed but mainly just twinges nothing that I can’t manage |
|  |  | She’s been the best Dr I’ve ever had, she listens to me and she asks me what I want you know |
|  | R016 | It is painful mind, god theres days where I have a boiling hot water bottle against my stomach and just in agony |
|  |  | I just thought straight away I’m going to have to have surgery again, I’m gonna need time off work and can I afford that you know [pause 3 seconds] thankfully at that point me boys were abit more grown up |
|  | R017 | The medication they gave me for my Crohn’s flares up my psoriasis, so erm its been difficult to try and find a drug that treats both [pause 3 seconds] which is extremely frustrating as my Crohn’s stops me from going out and socialising and erm just having a life basically but when my psoriasis flares up [pause 2 seconds] its so bad it hurts to put clothes on so again I have the same problems so its been, its been abit of a rollercoaster and one that I’m still on |
|  |  | Its probably bad but I just use the internet to look up things, and I know, I know you only see the horror stories |
|  |  | I think what was more annoying was that id spent years studying to get on to the degree programme and then that was sort of taken away from me [pause 5 seconds] but I still finished, I still finished |
|  |  | I’m often trying to eat more of a well balanced diet, erm get regularly sleep kind of at least 8 9 hours every night and also avoid alcohol |
|  |  | When I have a flare up my feet used to swell up and I used to get bad joint pain around my ankles [pause 2 seconds] so that wasn’t pleasant and also erm made it difficult to do the things I wanted to do |
|  |  | Abdominal pain it just comes, sometimes there’s no easy explanation as to why it’s come on [pause 2 seconds] erm some days I’ll be absolutely fine and then I have a day where I’m really bad. Erm when its joint pain its normally related when I’ve had a bad flare up, erm [pause 3 seconds] I haven’t really worked out when and why erm I get it I think its still a learning curve with that one |
|  |  | You need to make sure that you rest and get enough sleep, I don’t think there’s anything I do particularly to reduce my pain. I’ve tried the drugs, heat and stuff but when it comes you just kind of just have to wait until it passes [pause 5 seconds] I think I’ve spoke to you about it before but I suffer from severe erm fatigue [pause 4 seconds] and I always think getting enough rest can make you feel better erm as I mentioned before the interview work are really good with my fatigue and pain and erm are more than happy for me to have a nap on my lunch break |
|  | R018 | I think it would usually be diet or food related. I blame myself because I’ll eat something and sometimes I know its bad but I’ll eat it, so that can be a trigger. Sometimes it can be totally random though, I’ll just have a bad uncomfortable day and the next I’ll be better, its kind of weird which is why I get abit concerned about stuff I have upcoming in the future like work meetings, events things like that if you don’t know what sets you off fully and it takes a while to get in to remission |
|  |  | It was very mental exhausting seeing people your age go off and celebrate finishing uni and getting jobs and your stuck in the hospital or at home because of this [pause 4 seconds] and like erm you loose friends, you stop getting invited to places or events because you aren’t well and that takes it toll |
|  |  | I think some [friends] try to understand, you get the ones that look at you and you can just see they feel sorry for you and you get the ones that just don’t and will never understand [pause 2 seconds] whether that’s helped by me not telling them the real reasons I don’t want to go out or do things is actually because I’m too tired to |
|  |  | If someone said to me I’m tired id say to drag yourself out it will make you feel better and the other thing is I don’t want to have to say I need to be close to the toilet in case I need to urgently go [pause 3 seconds] its just not worth having to go through that so you just don’t bother going |
|  |  | Sometimes skin type conditions so like psoriasis and other skin disorders I get are quite common. Which could be related to taking azathioprine for a long time and having a low immune system |
|  | R021 | I do find being at home sometimes like a prison cell [pause 3 seconds] which might sound stupid but your confined, I don’t know If that’s the right work but your stuck aren’t you [pause 4 seconds] I think if I didn’t feel trapped erm, like stuck and was able to leave the house and not worry about using the toilet or finding a toilet then it might be erm different |
|  |  | With the tummy pains erm it can be with food but yano its hard because I could just have a stressful day and that will trigger it then next day I’m ill [pause 2 seconds] you never know when your gonna have a good day or bad day but you just got to get on it’s the cards I’ve been dealt so have to deal with it |
|  | F002 | I do find if I am stressed that I tend to use the toilet more and the urgency sometimes comes as well [pause 2 seconds] but staying near a toilet is fine and it’s something you get use to |
|  |  | I haven’t really found anything than taking pain medication helpful |
|  |  | AS gives me the joint pain but then its hard to know which one is which that is causing the pain |
|  |  | I also have ankylosing spondylitis which I had before I had crohn’s, but they told me they were both linked [pause 2 seconds] although I don’t know anyone else with AS so mustn’t be that common [pause 3 seconds] but to be fair I probably have more problems with the AS than I do the Crohn’s |
|  |  | It was the azathioprine I was so ill with that it was worse than the disease and I, I remember thinking is this it, head in a bucket being sick not going to work |
|  | F007 | My injection is due tomorrow and I find the day, the day before my injection I’m really tired [pause 4 seconds], I get really anxious about going out because I’m scared I’m not gonna be well [pause 3 seconds] It’s not like I can ring people anymore and say I’m tired can you get me they just won’t understand and my parents are getting older so I find it best to just work from home or take the day off |
|  |  | I find obviously when my humeria is due that my anxiety levels, I’m not really you know not really in control of those when the injections due. |
|  | F008 | I think you loose all your dignity having Crohns [laughs] |
|  |  | Obviously fatigue is one of the biggest erm ones that I’m dealing with but I do have a young family so sort of pin point it to that rather than, than obviously the Crohn’s. I just don’t have the energy like I used to in order to er do stuff |
|  |  | I have a circle of people who have kind of got similar conditions and you know, er, kinda you know they and they spur you on you spur them on, on tag team. However, erm sometimes you’re a bit isolated with the condition not being able to keep up with people or not going out |
|  |  | Its mainly the fatigue I suffer with just feel like it’s a battle between my mind saying you can do it and my body saying you can’t |
|  |  | I’ve tried through food to try and manage it that way, obviously I don’t drink alcohol, erm tea, coffee, well tea I don’t drink any more because again laxative and also it stops nutrients being absorbed into the body but erm I only learned that through reading online |
|  |  | You erm don’t really get told much about things you should avoid like I love tea but I’m always feeling tired and then I was reading online that erm tea stops iron being absorbed so maybe that’s why I was feeling tired yano. Obviously coffee does kind of similar but not quite as bad and erm erm just effectively being more, more mindful, having said that because obviously your fruit and veg you know aggravates it |
|  | F009 | I’ve got bad bones as well, but what did they expect I was on steroids for over 5 years. |
|  |  | I was also put on steroids because of the pain in my tummy, obviously the information on using them wasn’t really known then and that’s why I think I’ve had so many problems |
|  | R024 | You know its been worse so there’s no point complaining about pain now when its not as bad as it was |
|  |  | I thought I was indesctrubale until the condition and all of a sudden your like, well can’t do that anymore |
|  |  | They got it under control and it behaved for about eight to ten years but then it just came back with a vengeance, it was in such a state I had another fistula, I had two more resections and nothing was helping so I ended up getting my bowel removed and an ileostomy in |
|  |  | Abdominal pain I’ve had that since before I was diagnosed, I think its like everyone though there’s worse days and better days I think it just depends on what I’m doing, what I’m eating and how I feel yano in myself. Think stress doesn’t help it |
|  |  | The best thing I’ve found is turmeric, I eat it all week now. I didn’t think I would tolerate it very well initially, but it was recommended to me by someone else with the condition so I thought I had nothing to loose and it’s been great |
|  | F015 | I don’t think I’ve slept properly since before I was diagnosed |
|  |  | Me joints did swell and do swell when I er go through a rough patch but it’s never stopped me from doing stuff like the stomach cramps most the time its alright but there’s days I’ve had to take off er but I’ve been lucky last few years |
|  |  | I don’t know if that’s a thing with Crohn’s though, but the stress of having the condition while self-employed probably er hasn’t, it hasn’t helped. It also maybe the reason why I flare up quite often, not being able to manage stuff [pause 3 seconds]. |
|  |  | In the past I’ve been doubled over in pain, its been crippling you know. Cos it’s hard to describe because what you see as something being bad may not and you know the opposite, which is why I don’t really like to tell people I’m in pain because they either just assume your being a wimp or being overreactive, overreacting |
|  |  | Cos me flare-ups would happen I would be just basically, there was no talking to us, me stomach would be really tender and erm, sore on me right side where normally me Crohn’s flares up. There was just really severe bloating so I just put it down to constipation. It just, first time in maybe 3 years you know what I mean I’ve had it so, but then it basically passed. I think it was a combination of the fact I had been eating erm, cashew nuts and bananas erm and I think it was just a combination of things |
|  | R027 | Joint pain in my fingers I getting worse, but I think that’s because I’m doing quite a lot of gardening and doing more stuff were I repetitively use my hands so I think that’s why. But I guess it does stop me from doing that until it eases off. |
|  |  | Recently I’ve noticed the cold hurts my hands as well as I’ve just had to go out an buy some gardening gloves and handwarmers |
|  |  | [Abdominal pain] Sometimes a warm bath or paracetamol help [pause 3 seconds] or a hot water bottle but nothing really properly helps you just have to wait until it passes |
|  |  | I went to go and see a homeopathic doctor, erm and I also had sort of massages and things like that with rheumotherapy oils, the stuff with the homeopathic doctor did seem to work very well but I don’t know whether that’s because I wasn’t allowed to eat much at all [laughs] there was a lot of things I couldn’t eat which is one of the reasons I stopped because I started getting deficient in things but erm, [pause 2 seconds] I’ve not really found anything else as effective to be honest with you |
|  | R028 | I try not to do too much and even when I don’t I still feel as tired as what I would do if I had a busy day you know [pause 3 seconds] and I, and I [pause 3 seconds] I look after people, you know for a living and I can manage but I can tell when my body is due the next lot of humeria, it can like stop my periods and I need a few days off before I get it because I just can’t manage [pause 4 seconds] but that’s obviously with the condition |
|  |  | Oh I get total fatigue with it, like tiredness its really hard to explain because like you say to people I feel tired and they just say go to sleep, go have a nap [pause 3 seconds] but it just isn’t like that |
|  |  | Making sure that im not eating rubbish all the time. When I get fatigued its just a constant battle between my head saying do it and my body saying I can’t, so eating convenient food is my go to |
|  |  | I do actually get aching joints but when I’m about or in a flare up it’s a good indication |
|  | R030 | A warm bath used to help the pain but I don’t do anything for the pain I get now as its manageable and doesn’t interfere with my day to day activities [pause 3 seconds] and to be honest I’d rather not take medications unless I’m in a bad way |
|  |  | I didn’t have it like I said I wouldn’t have any sort of life, it was a battle that I was loosing |
|  |  | Think you have to admit defeat and know the tablets aren’t working and to have a better quality of life something needs to change |
|  | R031 | I know what triggers it if you know what I mean |
|  |  | I would probably avoid some spicy foods and have something quite bland but that was pre surgery |
|  | R032 | It had complete control over me, what I did, what I ate, where I go or in this case didn’t go, it’s like I was completely powerless. But now my goodness I’m living a whole new life and I think it makes you appreciate the things that you normally would just take for granted |
|  |  | Your like symptomatic the drugs make you ill and fat and your in pain it’s not a combination you really want. But over the years you just get used to it and take the good with the bad |
|  |  | Have a few bad turns when I was allergic to some of the drugs that were for Crohn’s but they managed to find one that fitted and since then touch wood I’ve not had a bad flare |
|  |  | When you have a flare up you just have to make yourself as comfortable as possible and wait for the doctors to do their thing |
|  |  | It was actually one of the reasons why I decided to become self employed because I had more control over my sick days and to be honest the thought that I could loose my job because I wasn’t well probably stressed me out even more so definitely was one of the main reasons I started my own business and you know I get help from my wife and family |
|  |  | I was on very high dosages of steroids which made me put weight on and I was in a really bad way so much so that I wanted to have my stomach ripped out it was awful |
|  | R033 | I was admitted to hospital with an acute small bowel obstruction which initially they thought was related to my Crohn’s but it turned out I had a tumour in my abdomen and I had Hodgkin’s lymphoma which I know from the literature can be associated with the use of azathioprine so they stopped that and I haven’t been on anything since and id like to keep it that way as I feel well in myself |
|  |  | I think just being able to do the things I’d done prior to being diagnosed, doing them without feeling tired, thinking about the location of the toilet, carrying medication, yes probably not having the urgency and being up during the night needing to use the toilet and I would say feeling like you can socialise with friends and it not take it out of you. I think those are the things you do take for granted and are perhaps not considered when treating the disease |
|  |  | Abdominal pain sometimes but I’m not sure if it’s the Crohn’s as I do have a lot of scarring in my abdomen |
|  | R034 | I think when people call in sick with a cold, id like come in if I had a cold because I want to save me sick days really for when im actually ill with my Crohn’s |
|  |  | I find sometimes that the disease is more mental than physically draining |
|  |  | Well erm they gave me energy don’t get iz wrong but erm they changed my body and I ended up putting on loads of weight so like erm for example I didn’t go out because I needed the toilet all the time and erm I wasn’t like well [pause 3 seconds] then the steroids made me well but changed my appearance and so I didn’t want to go out again [laughs] just didn’t go out. Wasn’t until the infliximab that I felt like I had my life back abit |
|  |  | Joint pain I get in my knees and hips, its fine most of the time but when its bad its crippling [pause 2 seconds] like I cannot walk and that’s not helpful when I work in retail and am on me feet all day so it has made me have to take days off |
|  |  | I can’t have like spicy foods and that, I do avoid them and if I find a food that I think flares it up then I avoid it [pause 3 seconds] erm what else, its hard to think really as I think you just get used to doing the stuff and not realise you erm stop them |
|  |  | I used to get flare-ups quite often but thankfully they have been better since the infusions erm but they take a chunk out of your day and your life [pause 3 seconds] I know some people have it worse when they get er drips all day and mine are only for a few hours every so many weeks its not bad |
|  |  | The like people on the forum they go through so much with needing IV drugs all the time, I’m on nothing so how I can I complain when they are experiencing that, you just can’t you know it makes you think how lucky you actually got it |
|  | R036 | Never really thought about joint pain as I just put that down to age [laughs] never really thought about it being part of my Crohn’s |
|  |  | It manages you and responds to how you feel in yourself, so if your stressed it will respond if your relaxed and calm it will be as well so I think it does rely on how you feel in yourself |
|  |  | I was on the mercaptopurine and the azathioprine my erm life wasn’t to great, you had an existence rather than a life that’s what my wife says about it |
|  | R037 | It wasn’t a very easy time not with children when my husband worked full time, I really struggled to get back to normal and I think that was because I erm was so poorly beforehand and I just lost so much weight, I didn’t even recognise myself but I knew I needed to do it |
|  |  | The abdominal pain has been throughout erm, don’t get me wrong its not bad all the time just niggly but it has been there ever since I can remember |
|  |  | It’s just rest and not to eat, when I do have the abdominal pain I try to just take on fluids than eating food |
|  |  | It’s just constant lethargy, constant tiredness and it doesn’t seem to matter what I do whether I try to sleep or rest or be active it it doesn’t make any difference |
|  |  | Don’t get me wrong, don’t get me wrong I’m not ill [laughs] I don’t feel ill as such they are just niggly little complaints that you sort of cope with on a day to day basis |
|  |  | I’m careful about what I eat, erm I can’t over eat as soon as I feel as though I’ve had sufficient and don’t get hungry pains, I can’t eat anymore I won’t eat anymore because I think over eating erm can exacerbate the problem erm so [pause 2 seconds] I tend to try and eat smaller amounts but regular [pause 4 seconds] |
|  |  | I think the food makes it worse because I think I’m giving my stomach a [pause 2 seconds] a lot of work to do If that’s the right term [laughs] and erm by I think resting it and just drinking fluids and having crackers, I’ll live on cream crackers and toast, dry toast for a while until it settles down and then I can go back to eating normally. I mean I eat quite a variety of things but there are certain foods I try to avoid |
|  |  | They tried to put me back on something but I’d rather not take anything as I get terribly bad headaches with them or nausea so you end up in a catch 22, but those symptoms are common from what I’ve read online |
|  | R038 | It wasn’t just the, going to the toilet the pain that took the years away from me it was side effects from the medication and the other symptoms that you just can’t treat, the tiredness |
|  |  | It’s difficult because is it also linked to the fact I had a less stressful week or had a better weeks sleep so it’s hard to distinguish but I think you get used to feeling constantly tired |
|  |  | Now I just get the fatigue which is always there, I’m just a constant walking zombie my brain doesn’t fully function |
|  |  | I’ve tried things like pacing abit with the fatigue, but other than that [pause 3 seconds] not really I haven’t found anything that helps |
|  |  | I’m just constantly in a fog, honestly sometimes I would prefer to have the pain or the diarhoea because you know that goes away |
|  | R039 | Since having the stoma formed getting on for 20 years ago, touch wood I’ve not had many flare ups |
|  |  | Abdominal pain, before my surgery I’d just get on with it nothing was touching that, I’d take loads of pain killers and it just not do anything, hot water bottles sometimes helped but er you just had to wait until it passed. Sometimes I was rolling on the floor with it, other times I could get on with my activities so it varied on how bad it was |
|  |  | I think before my surgery I didn’t have that option to try things as I couldn’t keep water down I was so ill so it’s only after the surgery I’ve been able to try different things. |
|  |  | The forming of the stoma which was the only option at the time I couldn’t keep water down I was so ill |
|  | F018 | I don’t get abdominal pain at all now, I’ve rarely had that since the surgery and its only been when I’ve had two little sort of flare-ups |
|  |  | I was still always very tired all the time sort of just general like lack energy things like that but not bad compared to the actual symptoms with Crohn’s and erm I would say that’s probably the main thing now for me is just the tiredness more than any problems with the crohn’s disease [pause 2 seconds] but you just get people saying your tired because you have kids, don’t get me wrong I am but I know what fatigue feels like, its overwhelming and nothing can really help |
|  |  | I’ve tried lots of other things as well erm [pause 3 seconds] but I, I don’t think there’s anything out there that’s like a magic cure at all [laughs] well not that I’ve found. But there’s no over the counter medication for tiredness or fatigue its just constant and I haven’t found anything that particular helps yet and I’ve had the disease over 30 years |
|  |  | Occasionally I get sort of wrist and knee pain, sort of like they feel just like achey, but then I’m sort of 53 so you don’t know whether its down to menopause now as well so it’s hard to know what’s Crohn’s and what’s just generally from getting a bit older |
|  | F019 | I guess I always feel bad on friends who have come out and then you need to leave [laughs] plus I erm, I probably wouldn’t go out with friends if the urgency of going to the toilet was there as I just feel as though that’s erm, its not erm something I want to share with them or something I would even want them to try and understand [pause 3 seconds] so I guess it just depends on what symptoms have decided to show up during your flare up [laughs] |
|  |  | I just take sometimes just like a ibuprofen or just over the counter painkillers yano if its really bad, otherwise just get on with it |
|  |  | With me azathioprine it sort of suppresses your immune system so I’ve got to be really careful for like bugs and colds and flu’ and stuff so it is a lot more to take on and be a lot more cautious to what I’ve got to be round |
|  |  | I had to reduce them [steroids] down until I stopped taking them, made iz gain loads of weight, helped my bowels but not what you want before your mate gets married |
|  |  | I take some paracetamol just to see if that eases it. Sometimes it does, but other times I might put a little hot water bottle on it to try to ease it |
|  |  | I’ve just started taking like peppermint tea as well to try and like reduce the bloatedness |
|  |  | I do get a little bit of pain in me joints like the lower part of me back. I didn’t realise that, that kinda alongside me Crohn’s as well, I just put it down to a long day at work until obviously all this is came together with us having the Crohn’s and like me consultant and people are saying you have to be careful |
|  | F020 | The abdominal pain eased of after the very last bit of surgery, yes, er I just kept getting bowel blockages so I’d sort of be in agony with that and the only thing that would help was morphine |
|  |  | If I’d had a stressful day or if I did something out of the ordinary that put my body under a certain pressure I definitely noticed the pain and bowel habits changing. Perhaps some foods, spicy food particularly but I just stopped having them, wasn’t much of a fan of them any way thankfully |
|  |  | I’ve tried aloe vera, and erm, I tried experimenting with diet, um, and nothing seemed to make any difference particularly |
|  |  | I’ve had a severe attacks which have unfortunately led to my bowel essentially no longer working, and because it no longer functions I do require nutritional feed. So I need to get my bloods checked on a regular basis and then my food bags are made up specifically to what I require. Erm, I do also have a few issues with my kidneys and liver because of the feed |
|  | F021 | If I am particularly stressed at work it does have an effect erm been on supplements in the past if I’m feeling tired and run down and things erm but erm since starting the new medication I’ve not needed to have that, so I just try to take a multivitamin to try and sort of keep that balance and sort of keep things relatively normal |
|  |  | It’s always just been the abscess or the strictures yeah [pause 2 seconds] they just seem to reoccur and then it ends up with me needing surgery |
|  | F022 | So I find it’s a battle, it’s a psychological battle as well as a battle with the disease of Crohn’s if you want to call it that. |
|  |  | There’s days when I’m very tired but most of the time I just get on with things. |
|  |  | I just get really bad trapped wind sometimes since starting this new immunosuppressant which has increased my bowel habits so will just see how that goes |
|  |  | I get occasional skin complaints, erm, psoriasis on Monday which has now gone and joint problems and that’s it. |
|  | R041 | I think I just found it difficult to tell my boss, oh I can’t come to work because I’m scared I’m going to have an accident on the bus on the way there |
|  |  | I could just sleep and don’t feel any better |
|  |  | My family noticed, my friends noticed and even my work colleagues started to see a difference [pause 2 seconds] you can only put on a front for so long |
|  |  | The pains in me hands and feet has gotten worse over the years so I’m getting referred for that |
|  |  | I started on one tablet he gave is and that was making is poorly erm me hands very sore erm so then I went on azathioprine, I was doing alright with that erm but when I had a [pause 3 seconds] erm erm, they did a liver function on is and it was causing damage to me liver so I then went on the infliximab which I was doing okay on that but then I had another flare-up |
|  |  | For my joint pain, at work I erm I don’t have a mouse I have a roller bar so its like, its like an attachment that goes like from the keyboard so rather than clutching onto the mouse I just have to roll me hand, roll me hand over it and that does help a lot as obviously I work on the computer every day. Erm what else, maybe sometimes like a hot water bottle, pain killers but erm normally just waiting for it to pass and just getting rest |
|  |  | Think that’s the worst part about it [pause 3 seconds] I don’t drive, I get a bus to work which takes me 40 minutes and there’s no chance, like no way I can wait 40 minutes. So it made me feel extremely stressed out and anxious, which you know I think makes you worse but erm [pause 3 seconds] erm yeah I couldn’t travel to work and had to take a while off because I was quite anxious to travel |
|  |  | Since I was diagnosed with the abdominal pain its mainly there when I flare up, I don’t really get it when I’m not flaring up or maybe niggles but nothing that I can’t sort of handle |
|  |  | The looks you get when you ask someone to jump the queue at the toilet, yeah not worth it, or or when they say you look fine and your like well do I explain what I have it is difficult |
|  | R044 | The joint pain I don’t know whether its my AS or the Crohn’s but they are both related [pause 3 seconds] its just hard to tell them apart |
|  |  | Just trying to avoid doing things like repetitive stuff and getting plenty of you know rest and I guess just trying to not let it get in the way [pause 2 seconds] but it does stop me from doing erm things |
|  |  | I tried a few different tablets but found I was intolerant to them, I think it was, I can’t remember what the exact problems were but I think they were about liver function and that sort of thing so I only lasted on those for a few weeks |
|  |  | I’d wake up, get ready and head straight back to bed and even then sometimes I’d wake up worse and you know that’s always kind of been there the low energy levels but I’d always put it down to getting abit older and thinking this is just what came with it. |
|  |  | I’ve sort of got into a routine where I know what to eat and what not to eat erm which works 80% of the time [pause 2 seconds] but don’t get me wrong I get it wrong now and again and I still stray off the diet now and again and just suffer the consequences |
|  |  | Probably a good diet, the one thing I never got mastered and I think it’s a big factor, but I haven’t got this mastered yet, I think well it’s a personal thing, I think hydration has a lot to do with it erm, so I work in a job where [pause 3 seconds] I forget or maybes not able to keep the hydration levels up erm [pause 3 seconds] and if if I have horrific pain, I make the concise effort to drink and drink and drink, and I do feel better without a doubt |
|  | R045 | It’s worse sometimes and better others, which I think its why its so hard to take tablets because if its only going to last a few minutes then I don’t really want to be taking codeine or tramadol which will make me drowsy [pause 2 seconds] so you put up with it |
|  |  | I found it harder when I’ve stopped smoking, I find it, I find if I’m having a flare up its generally from something I’ve eaten just like cheese, sweets like starching stuff. |
|  | R046 | It’s quite hard to really, to distinguish ‘cos I’ve had so many different things that really, the Crohn’s disease to a certain extent has been the least of me problems |
|  |  | I’d take everything I had and still be in pain. I’m talking codeine, morphine, tramadol. But after my last surgery everything seems to be okay |
|  |  | Probably just watching what I eat. I tend, or try to avoid fatty foods, things like pies and pastries which are my favourite |
|  | R048 | I would say I get joint pain, mainly in my fingers but its hard to tell whether it is the Crohns or that im getting abit older [laughs]. I know its related to the condition but I’ve never been treated specifically for the joint pain, I guess thinking about it now its not something that you get asked about at appointments its always bowel related queries |
|  |  | I don’t think the join pain started for a few years after now I think about it [pause 4 seconds], but then again I think when your getting abdominal pain you don’t think about other pain your getting just focusing on what is worse |
|  |  | The cold sometimes makes it worse or if I’m gripping something for a long time but its nothing where I can’t stop it whatever I’m doing and it eases off |
|  |  | I have had a few bouts but its hard to establish whether it is Crohn’s or whether its side effects from some other drugs I’m taking |
|  |  | I would say just certain foods make the abdominal pain worse, but I tend to avoid what I know aggravates it |
|  |  | It’s really there’s not right or wrong reason as to what I can eat, its just trial and error really, I mean anything with a sauce in it seems to cause havoic and we think that might be something to do with flour or butter. But that could lactose intolerance I don’t know |
|  |  | I used to get the abdominal pain before my surgery but not so much since the stoma, the odd twinges I would say and again if id eaten something that it didn’t agree with. But I think after the surgery things started to ease |
|  | F023 | I don't know if I've told you before all the medications I've been on, but I've been pretty much like I've always had some kind of reaction to them |
|  |  | The abdominal pain its 80% just more of an irritation that maybe doesn’t last very long and is sometimes eased by going to the toilet. So apart from the hot water bottles, maybe sometimes just pain killers, but to be far it has been much better since I’ve had the hysterectomy |
| Resilience | F001 | It's always been a mental struggle because of my experience all the way through Crohn's. If you are if you are mentally stronger than you become, physically stronger, you know. Yeah. And vice versa as well, vice versa. The more physical strength you have, it improves. It makes you feel better about yourself. |
|  |  | I started doing a lot of fishing since my operation, and in the last two years and a lot more exercise which I think has just helped me manage my stress levels |
|  |  | Just from experience, obviously, eight years ago, I have going through stressful situations at work, I have stressful situations at home, I felt like I didn't have an escape, you know, when I was first diagnosed as having nowhere to go to to break that cycle, you know, so it was pretty bad at the start. But I think as you get older and I've matured, I can deal with things a little bit. When I was younger again, so I can control the stress and I can control the things that Mr. Mega stressed. But it's not that easy, you can’t just turn off the emotions and the stress |
|  | R002 | I’ve done a lot of reading up on it or whatever and a couple of books I’ve read about erm diet especially sugar erm and carbohydrate erm doesn’t do anything for IBS or IBD so I have, erm, I was trying watching me weight whatever and now erm I’m on a bit of er, a Mediterranean diet now so er, high protein with very little carbohydrate |
|  |  | I do think diet is really important, but you just struggle with a er, erm bowel condition that’s effected by food [laughs] you know, you know its hard as I love having nuts but yeah they hurt like hell [laughs] so its hard to get the right stuff in you without it hurting or needing to use the er loo and erm yeah you then need tablets because you aren’t er getting enough of the oh what are they, the erm, erm the b12 injections like stuff. |
|  |  | I found a book online about people with other gastro conditions not just like Crohn’s and that suggests cutting out sugars to help with the pain and help things flow [laughs] but yeah I sort of just try and follow that |
|  | R005 | well fruit and veg, and that kind of does make it worse, but nothing that [pause 2 seconds] I haven’t been very good at keeping a food diary to try and work things out erm [pause 3 seconds] I don’t eat anything wholemeal [pause 2 seconds], I should really [pause 2 seconds] oh actually recently I have noticed anything fried tends to make me worse, so I don’t eat a lot of fried stuff or abit oily I notice a difference, yeah [pause 2 seconds]. I have also thought about giving up diary [pause 2 seconds] but I haven’t done it I’m just not sure if it will make a difference I need to probably keep a diary and see better [pause 4 seconds], but erm, just, erm just mainly because I don’t have the time and don’t have much hope in it like realistically how much are things going to get better |
|  |  | The longer I’ve had the condition the more I feel like I can manage it myself |
|  |  | I guess I always feel bad on friends who have come out and then you need to leave [laughs] plus I erm, I probably wouldn’t go out with friends if the urgency of going to the toilet was there as I just feel as though that’s erm, its not erm something I want to share with them or something I would even want them to try and understand [pause 3 seconds] so I guess it just depends on what symptoms have decided to show up during your flare up [laughs] |
|  | R008 | I avoid roughage, that’s all I’ve ever been told really is to avoid roughage [pause 3 seconds] I can’t eat, I mean as much as I love cabbage I can’t eat cabbage, erm a lot of fruit I can’t eat. I did have oh a couple of years ago I had a coconut cake and it caused a blockage, but you know I just avoid things you get used to avoiding certain foods don’t you |
|  | R009 | People think you look fine and its so frustrating, after I had my stoma I think it changed my way of looking at things. I don’t care about it, its great i tell everyone about it primarily to try and educate them. |
|  |  | It can affect my day, I just to try, I get on with it whether that’s right or wrong I don’t know, but I do |
|  |  | I was on between 2013/2014 a no residue diet, or a low residue diet so I went from very, very horrific diet to then eating like a queen |
|  | R012 | I think I’ve been quite lucky with that [pause 3 seconds] my flare-ups would tend to be [pause 2 seconds] I’d be on the loo 5 times on a morning sort of thing, but it’s never really stopped me erm particularly doing anything [pause 2 seconds] I mean I may change my plans slightly if I’ve had a bad morning I may only go out in the afternoon or something like that, but I’ve never been so bad |
|  |  | I’ve never needed any hospital treatment or anything like that so I’m lucky in that respect [pause 4 seconds] I feel guilty to be honest saying I have the disease because I don’t experience the symptoms |
|  | R013 | You go through the ups and downs with the condition, it was difficult for me in school having to explain why I was off or back and forth, kids are just awful but after a while id tell them and in some occasions it actually made things better |
|  |  | I’ve not been the best with diet [laughs] I have tried different diets like soya milk but I wouldn’t say Ive tried anything properly where I’ve kept to it [pause 2 seconds] I think I just feel as though I don’t want to stop eating or drinking the things I enjoy |
|  |  | I guess because I have had it so long I maybe don’t find myself nerved by things anymore |
|  | R015 | I’ve just been careful with what I’ve eaten. I couldn’t eat like nuts ‘cos they always upset me stomach, I couldn’t drink wine and nuts I would be very poorly. I tried to like control it through diet |
|  |  | You think you’ve been through something terrible and people wonder how you’ve managed, well you know you don’t have a choice you get on or you give up and let it em become you but yes you have these terrible bouts and then something else worse comes along or something worse happens and not just necessarily with the Crohn’s but things that could be associated with the Crohn’s. It doesn’t rain it pours I think that’s the saying for it |
|  |  | I just think because I’ve coped it with and its my coping mechanism to see the condition the way I do em, but it’s really good to see that I can do things to help myself |
|  |  | I need to know I’ve tried everything before giving up |
|  | R016 | I don’t think ive personally had that much of a rough ride with it [pause 2 seconds] not compared to others you know but I do think, I do think that there needs to be more information given about all the things you can get |
|  |  | I know what certain foods that I cant have but other than that I try to eat healthy and drink water what else can you do really |
|  | R017 | I just used to be very careful with me diet but now since I’ve gone onto the infliximab I can just eat anything I like at all. But again be careful with nuts, nuts are the main thing I would avoid nuts because I would be guaranteed to have very loose bowels the next day. Oh I also find seeds in or on bread to be the same which you know is difficult because if you are at an event most places have this exotic bread and nuts in things and you end up not having any of the food anyway in case you feel like its going to make you go to the toilet |
|  | R018 | I’ve went to homeopathy before, I’ve erm tried reflexology, I’ve tried supplements, |
|  |  | Other than that the usual tiredness, I do work full time which I think it takes it out of me much more than a normal person erm and the main thing I think mood is important as well feeling abit exhausted leaves me feeling or having depressed feelings from time to time erm but yeah you just power through I guess |
|  | R021 | If you can’t laugh at yourself then who can you laugh at, I mean we have nicknames in our house for my meds and my walking stick the grandkids love it |
|  |  | I just think you get use to taking 10 plus tablets a day |
|  |  | Stop eating things that make me want to go to the toilet, like fish and chips, oranges are alright but if i have an apple and it goes right through is. Just trial and error, 30 plus years of it which hasn’t been easy you kinda wish there was a List of things that was gonna hurt but its so different for everyone yano so just a lengthy process of working out whats works well and what like doesn’t’ |
|  |  | If you want something you’ll have it but you just have to pay the consequences, up all night on the loo or pain but its hard not to have summit when ya want it and other people have it |
|  |  | I think you just get like that don’t ya just fed up but I’ve had the crohn’s now for over 30 years and god I just think sometimes that if I didn’t have it things would be a lot different |
|  | F002 | Obviously just avoiding foods, but other than taking tablets which I don’t really like to do nothing much I found has really helped apart from sometimes if I have an upset stomach I try drinking a peppermint tea |
|  |  | Really I think compared to many people with the disease its fairly mild |
|  |  | Just things like nuts and seeds, they don’t sit well with me but I’m not really bothered about cutting them out [pause 3 seconds] if I know that’s what sets it off then I’m more than happy to not have those or have them in moderation |
|  |  | I do find if I am stressed that I tend to use the toilet more and the urgency sometimes comes as well [pause 2 seconds] but staying near a toilet is fine and it’s something you get use to |
|  | F007 | When I had the flare-up had to I had to eat things I wouldn’t normally eat, erm [pause 2 seconds] like wholemeal bread is obviously is healthy but you were put on white bread and things like that erm I did find like Indian food wasn’t great for me, like spices and obviously anything, [pause 2 seconds] sort of that food related isn’t good. So I guess I would [pause 2 seconds] guess I would just stop eating certain things erm but like your not told are you, its just a hit and miss sort of thing I mean I kind of erm just eat the same things as I know that is what I can have |
|  |  | Just sort of things to avoid but like that’s what you have to do I mean I know if erm like if I had an Indians id be on the toilet all night and the pain its like just not worth it [pause 4 seconds] the toilet becomes your best friend [laughs] |
|  | F008 | I would consider myself fortunate as like you hear some people who have it so bad so I guess I’m one of the lucky ones that has come out the other end |
|  |  | It’s always after I’ve eaten you know particularly food and think you’ve just got to run with it as and when it comes [pause 3 seconds] you have to adapt and just accept its there and hold on to the fact it won’t last forever |
|  |  | I have a circle of people who have kind of got similar conditions and you know, er, kinda you know they and they spur you on you spur them on, on tag team. However, erm sometimes you’re a bit isolated with the condition not being able to keep up with people or not going out |
|  | F009 | When you first start having these back, those days and it was a lot about diet, they thought milk was the problem to begin with. And I thought and I did try to buy a probiotic actable drink. They didn't do anything. I'm not a very good girl. I don't watch my diet like some people I know who eat fish and chicken and whatever other people don't want |
|  |  | I’m on those injections which have been really good in controlling my symptoms. Not very pleasant things but you get used to being jabbed |
|  |  | Stress goes to my tummy. You know, if I put in taken out of my comfort zone with regard to lose or whatever, it's my tummy that tenses up. But the other thing that I used to happen to me was, again, terrible pain in my tummy. And when you get pain you get stressed because its there, you can’t do much about it and that makes it worse |
|  | R024 | I mean with the condition you don’t have any dignity left if you know what I mean, something I could write a book about. But its how to get the job done and avoids all the other procedures that can be done, I mean hey I’d rather do stool samples than have one of those camera tests. But any one with the condition, anyone who has had it along time is very easy and happy to do stool samples because its part of having the condition, just like I dunno being lactose or whats the word I can’t remember, being allergic to something means you can’t have that its just something you get used to |
|  |  | Abdominal pain, I think I’m quite fortunate, compared to what I used to be like anyway but at the moment but fruit and veg doesn’t seem to help at all |
|  | F015 | It felt like a long process but I managed and coped and I got here in the end |
|  | R027 | I’ve had no removal or anything just a drainage [pause 3 seconds] again I’ve been quite lucky |
|  |  | I don’t think I have it that bad actually I went for years without a flare and only flared last year so I’ve been quite erm fortunate with it. |
|  | R030 | Sometimes feel like a bit of a fraud as I have no issues with food or anything and I’ve got no pain, long may it continue [pause 2 seconds]. I’m happy as I feel quite normal, whatever normal is [laughs]. |
|  | R032 | When im less stress I do notice my bowel habits do change so I think trying to reduce stress has been helpful and I seem to have less bowel cramps [pause 3 seconds] so I think it does help, plus who doesn’t like a day off work [laughs] |
|  |  | I try not to eat sort of peppers, cucumbers or spicy sort of foods |
|  | R033 | My management of Crohn’s disease is just dietary, I just try and avoid things that are very high with roughage like kale and beans [pause 2 seconds] I don’t eat things like baked beans or kidney beans anymore I try and avoid those [pause 2 seconds] I think changes like that I’m willing to change because they can easily be implemented into daily living |
|  |  | High fibre foods make it worse as well, say I eat a packet of dried apricots I would definitely have abdominal pain the next day so its best to avoid these. Id rather avoid them than cause a flare up and experience the things I have and experience the things I’ve seen within my line of work |
|  |  | I don’t eat as much, that’s about it really [pause 4 seconds] the less I eat the worse the pain is so during the times where the flare up is particularly bad or I have pain ill not eat anything and that can go on for days. I find I don’t get that hungry feeling much I just feel full and bloated without having eaten anything, its strange and its probably not the best for me to do but I don’t know what else I can do |
|  | R034 | I think to be honest there’s times you forget you have it coz you just carry on and get on with things |
|  |  | I’m still learning about it |
|  | R038 | It’s taken me a long time to establish what I can’t eat unfortunately its different for everyone so one leaflet doesn’t fit all |
|  | R039 | There’s times in the past where I haven’t made it and I’ve been out in public and it’s the most unbearable experiences I have ever had, it puts you off going out and causes this sort of fear of it happening again and this time will people notice, did they already notice, will I be with me friends or out for an event yano [pause 3 seconds] so id rather just keep it [ileostomy] than even go back to that being a possibility again |
|  |  | I think I came to terms that I had Crohn’s a long time ago and, er I think because I’ve been through a lot with it |
|  |  | Nothing surprises me with what they can do, what it causes and how much it impacts you really [pause 3 seconds] I think knowing, well sort of accepting what you have means it doesn’t come as shock |
|  | F018 | I’ve had the condition for a while now so I know what I can and can’t eat so I just avoid them to be honest because its easier that way [pause 5 seconds] but its taken me a long time to establish what I can’t eat unfortunately, its different for everyone so one leaflet doesn’t fit all |
|  |  | I think I’ve learnt with the disease is that you have to try and do what you plan to do and not let it interfere but [pause 3 seconds] that’s easier said than done sometimes [laughs] |
|  | F019 | My sister has the condition as well, well she has UC, and I mean she can’t work and ended up missing loads of uni, yeah, so I’m the normal one |
|  | F021 | I’ve just learned to live with the fact that on occasions you’ve got to run to the toilet and there’s nothing you can do about it |
|  |  | Generally I just deal with it and I’m sort of in that mindset it’s part of the disease |
|  |  | It is just a case of avoiding things erm yeah, I think it is just a matter of erm [pause 2 seconds] sometimes you can get away with it and sometimes you cant and its sort of erm is just using prior knowledge what I’ve done in the past erm there’s no one thing that I go to that sort of helps |
|  |  | There was some studies on cod liver oil so I’ve tried all those kind of bits and pieces of things [pause 2 seconds] but I think its just fighting against it its just trying to live with it and sort of I know what foods I can’t eat, I know I can get away with certain things but yeah with diet and trial and error really. I think initially after the first resection erm I was sent to, to see a dietitian and I went on a three month elemental diet so I was just on the drinks for three months and over a Christmas period it wasn’t wasn’t the best [laughs] so everyone was sat having their Christmas dinner and I was sipping my drink [pause 2 seconds] so we went through that process to see erm if there was celiac’s and stuff as well, thankfully it wasn’t it was jus Crohn’s |
|  |  | I think because I’ve had the condition for a while I know what I can and can’t eat and you just get used to limiting yourself with certain things [pause 4 seconds] but I think if you asked me after I was diagnosed or a year or so after diagnosis I wouldn’t say it had been successful because I didn’t know much about my condition yet so I suppose it is very varied depending on how long you’ve been diagnosed for |
|  | F022 | I do find if I am stressed that I tend to use the toilet more and the urgency sometimes comes as well [pause 2 seconds] but staying near a toilet is fine and it’s something you get use to |
|  |  | I’m aware of there’s certain foods I’ll eat that could aggravate me in the next day if I’m waking up in an emergency to go to the toilet. Hot spicy foods, unfortunately curries, Chinese food which I absolutely adore. The other thing, the other thing as well is erm, I’ve noticed that if I eat some fairly small amounts of bacon and stuff like that it’s fine, but if I eat a large amount of bacon or eggs in the morning I’ve had it, it’s pointless going out until I’ve gone to the loo ‘cos I know I’ll just get attacked and er once I’ve had that I’m fine the body does what it does and them er I’m, I still am |
|  |  | But you know you do have those moments where you are just like oh you know what I’m going to eat it and then immediately regret it |
|  |  | Maybe I’m quite lucky with it or maybe I’m erm, maybe I should take more time off work I don’t know but I just end up dealing with it. |
|  | R041 | Definitely when I cut bread out that helps is, and at the minute since June I’ve only had maybe one of two cups of tea but I drink peppermint tea now, I don’t drink normal cups of tea so I don’t have any milk, although I have milk in other things so I haven’t cut it out totally and wouldn’t say I would eat anything or having anything that didn’t have milk in, it’s definitely in my cups of tea that’s helped is [pause 4 seconds], the bloatedness, cramps I mean at first they were horrendous but now they are maybe just once or twice a month that I think oh my god I shouldn’t have ate that, yano the cramping in my stomach and stuff |
|  |  | I think you just got to adjust and just accept you have it |
|  | R044 | Whether that means not doing something one day but the day after that is ok to do and erm I suppose trying not to think of the negatives with the disease it can be easy to focus on them and let that get to you but yeah I guess that’s a way of managing as well |
|  |  | Rest and just probably a good diet, the one thing I never got mastered and I think it’s a big factor, but I haven’t got this mastered yet, I think well it’s a personal thing, I think hydration has a lot to do with it erm, so I work in a job where [pause 3 seconds] I forget or maybes not able to keep the hydration levels up erm [pause 3 seconds] and if if I have horrific pain, I make the concise effort to drink and drink and drink, and I do feel better without a doubt |
|  |  | That’s daily, just something I live with but nothing [pause 2 seconds] its uncomfortable and some places and times but nothing like it used to be its manageable |
|  |  | Acupuncture, Chinese herbs, those healing bracelets erm, I tried seeing a hypnotherapist I’ve tried everything |
|  | R045 | You just got to maybe watch what you eat and do it like moderation [pause 2 seconds] its not like you can’t have it its just its not worth having the problems [pause 3 seconds] like people put sun cream on because they don’t want to get burned its just like that, annoying when its things you enjoy but that’s life isn’t it |
|  |  | There’s a group on Facebook its called erm [pause 2 seconds] Crohn’s and Colitis Forum and when you read there what, what everyone on there is going through, I have nothing compared to them, literally nothing compared to them |
|  | R046 | I notice that eating those things makes my pain and frequency worse so but not I dont seem to have those problems. I mean don’t get me wrong if I want to eat something I know will cause problems I’ll still eat it but only if I know I have nothing on the next day you know |
|  | R048 | Painkillers, but you know even they take a while to work er, and even then you have to wait to see if they have worked before you can take other ones [pause 3 seconds] er, erm I think I just live with it now it hasn’t been unbearable for a while so er, yeah, er I guess just painkillers and just pushing through, when it passes, yeah |
|  |  | I still cant work out what makes my stoma worse, trial and error works but it gets difficult to work out whether there’s a delay in the processing or if its something that was just eaten but I guess after a while you just have to eat everything to give it a go |
|  | F023 | I’ve tried every food like I’d eat the same food constantly for months to see whether it caused any problems. Then I realised that garlic and onion wasn’t very good for and that’s what triggers it for me, so yeah I just avoid that, well as much as possible as everything has garlic or onion in nowadays |
|  |  | Alcohol I’ve noticed it doesn’t sit well with me so I just don’t drink anymore. It causes me quite abit of pain and loosens my stools as well so Its not doing any good so I made the decision a long time ago to either not drink at parities or events or to just drink 1 and stop there. But to be honest I don’t really enjoy it so its more than none than anything |
|  |  | Short run its annoying [laughs] having to restrict yourself but a lot of people do it and its not the end of the world. Think I’d do pretty much anything or try anything really to help with my condition, just because I know how bad it can get |
|  |  | Think a lot of people a lot worse off than me, and I think a lot of people are like really tired with it |
